# Quantitative proteome and phosphoproteome analyses highlight the adherent population during *Trypanosoma cruzi* metacyclogenesis

**DOI:** 10.1038/s41598-017-10292-3

**Published:** 2017-08-29

**Authors:** Juliana C. Amorim, Michel Batista, Elizabeth S. da Cunha, Aline C. R. Lucena, Carla V. de Paula Lima, Karla Sousa, Marco A. Krieger, Fabricio K. Marchini

**Affiliations:** 1 Functional Genomics Laboratory, Carlos Chagas Institute, Fiocruz, Curitiba, Parana Brazil; 2 Mass Spectrometry Facility - RPT02H, Carlos Chagas Institute, Fiocruz, Curitiba, Parana Brazil

## Abstract

*Trypanosoma cruzi* metacyclogenesis is a natural process that occurs inside the triatomine vector and corresponds to the differentiation of non-infective epimastigotes into infective metacyclic trypomastigotes. The biochemical alterations necessary for the differentiation process have been widely studied with a focus on adhesion and nutritional stress. Here, using a mass spectrometry approach, a large-scale phospho(proteome) study was performed with the aim of understanding the metacyclogenesis processes in a quantitative manner. The results indicate that major modulations in the phospho(proteome) occur under nutritional stress and after 12 and 24 h of adhesion. Significant changes involve key cellular processes, such as translation, oxidative stress, and the metabolism of macromolecules, including proteins, lipids, and carbohydrates. Analysis of the signalling triggered by kinases and phosphatases from 7,336 identified phosphorylation sites demonstrates that 260 of these sites are modulated throughout the differentiation process, and some of these modulated proteins have previously been identified as drug targets in trypanosomiasis treatment. To the best of our knowledge, this study provides the first quantitative results highlighting the modulation of phosphorylation sites during metacyclogenesis and the greater coverage of the proteome to the parasite during this process. The data are available via ProteomeXchange with identifier number PXD006171.

## Introduction

*Trypanosoma cruzi* is a flagellate protozoan belonging to the Kinetoplastea class that causes Chagas disease, an endemic tropical disease in Latin America^1^. This illness has taken on extra-continental dimensions because increasing numbers of cases have been reported in non-endemic regions due to intense migration, which has made its transmission possible by blood transfusion^2^.

The life-cycle of *T*. *cruzi* involve more than one host and different developmental stages on each one. Which result in the parasite being subjected to distinct environments, making its rapid adaptation in response to temperature variations or a decline in the available nutrients. In the triatomine vector, the epimastigote (replicative) and metacyclic trypomastigotes (infective) are the main stages of development, whereas in the mammalian host the main forms are amastigote (replicative) and bloodstream trypomastigote (infective).

The epimastigote differentiation into the metacyclic trypomastigote is known as metacyclogenesis, and this process occurs inside the rectum of the triatomine. This process can be reproduced *in vitro* under chemically defined conditions^3^, making it possible to obtain several intermediate stages during the process. Another advantage of this process compared with the *in vivo* assay is the ability to obtain a large volume of parasites for biological tests^3^ with preserved characteristics, such as infectivity and interaction with professional and non- professional phagocytic system^4^.

To understand the mechanisms that mediate *T*. *cruzi* metacyclogenesis is indispensable identify the molecular events that must coordinate within this differentiation process. The first example is the influence of the proline amino acid on the stimulus of the parasite metacyclogenesis. This molecule has been shown to be the only amino acid to sustain metacyclogenesis *in vitro* on defined condition with high yield and low parasite damage^3^. Thereby, demonstrating that the proline into a nutrient poor medium is capable of maintaining sufficient energy conditions for metacyclogenesis^3^.

One of the most important events required for metacyclogenesis progression of *T*. *cruzi* appears to be adhesion to the substrate. The importance of this event was confirmed by the low yield of metacyclic trypomastigotes obtained during the process of metacyclogenesis under agitation or in siliconized flasks^5^. The adhesion process and metacyclogenesis were then associated with the nutritional stress of the parasite because the addition of nutrients under the chemically defined condition inhibits the two processes^6^.

The differentiation process in eukaryotes has been centred on the phosphorylation of eukaryotic translation initiation factor 2 alpha (eIF2α), which prevents the translational process under stress conditions, this process is preserved from yeast to mammals. In *T*. *cruzi*, the overexpression of eIF2α followed by nutritional stress lead to translation inhibition and increases the rate of metacyclogenesis^7^. Later, the eIF2α kinase (TcK2) was identified as being responsible for eIF2α phosphorylation, which was controlled by the binding of a pro-oxidative haeme molecule^8^. However, the molecular events leading to the control of the haeme levels and the molecules that coordinate this process remain unknown.

This work provides a comprehensive quantitative phospho(proteome) of the metacyclogenesis process. In this analysis, more than 4,000 protein groups and almost 7,400 phosphorylation sites (phosphosites) were quantified, and these were found to be involved in a wide range of previously known as well as novel processes.

## Results

### Differentiation rates at different time points during metacyclogenesis guide the experimental design

**Figure 1:**
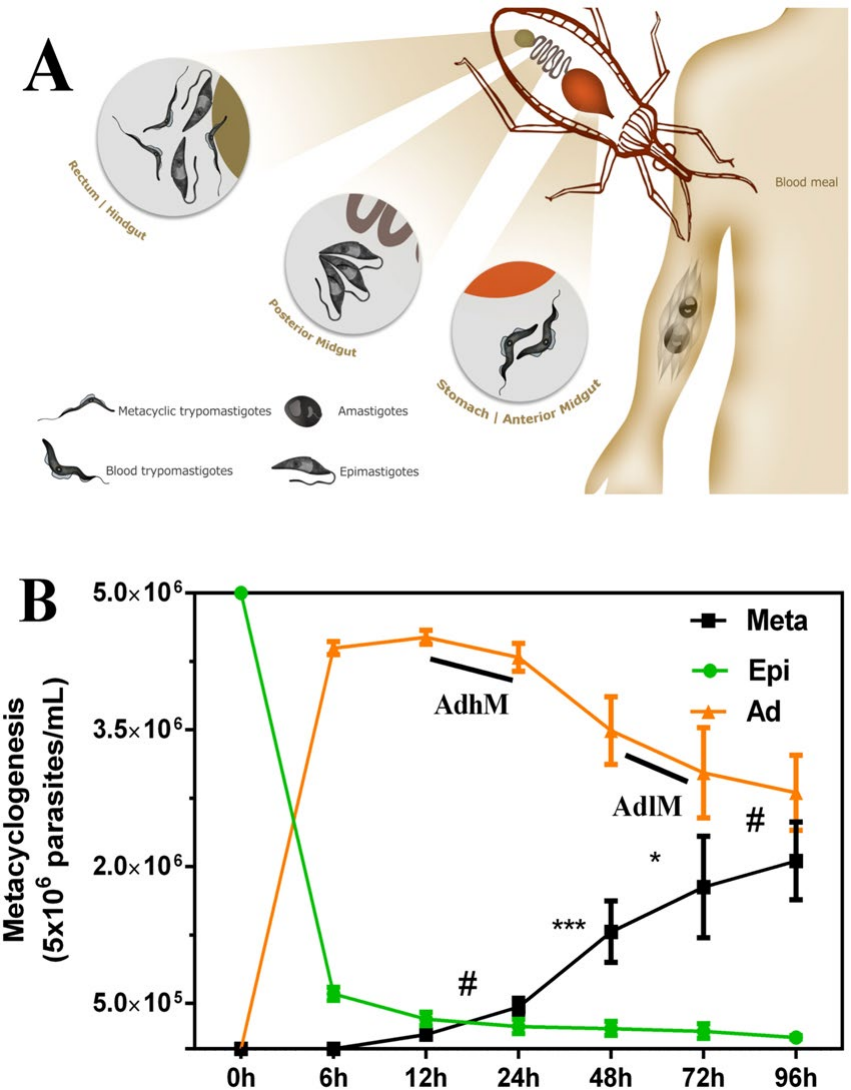
*In vivo* lifecycle highlighting the metacyclogenesis process and updated view of *in vitro T*. *cruzi* metacyclogenesis. (**A**) As contextualized in the Introduction, the parasite has two hosts. In its vertebrate host, the infective metacyclic trypomastigote form comes into contact with mammals through wounds or mucosal exposure, and the parasite then changes into its amastigote replicative form. After intense multiplication, it breaks the cells of the vertebrate host, and the blood trypomastigote form is exposed to the bloodstream. Subsequently, the mammal is bitten by the triatomine, and the trypomastigote blood form of the parasite undergoes differentiation into the epimastigote form in the posterior intestine of the invertebrate host. In the rectal surface of the triatomine the epimastigote form adheres and then differentiates in the metacyclic trypomastigote form. (**B**) For *in vitro* metacyclogenesis, after three exponential phase passages (3 × 10^7^ parasites/mL), the axenic epimastigotes were allowed to reach the end of the exponential phase (5 × 10^7^ parasites/mL). The epimastigotes were then subjected to nutritional stress in TAU medium (nutritional stress of 2 h) and later differentiated into the metacyclic trypomastigote form (6–96 h of metacyclogenesis). The number of adhered epimastigotes was estimated based on the total number of parasites subjected to metacyclogenesis (5 × 10^6^ parasites/mL) and their relation with the epimastigote and trypomastigote forms throughout the process. The AdhM form was the combination of Ad12 and Ad24h, and Ad48 and Ad72h were denoted AdlM. The data were statistically analysed by two-way analysis of variance (two-way ANOVA) and Tukey’s test for the comparison of averages (mean values ± S.D. from three independent experiments, each of which with technical duplicates). The symbols indicate the following: ^#^not significant, *p < 0.05 and ***p < 0.001.

The *in vivo* (Fig. 1A) and *in vitro* (Fig. 1B) metacyclogenesis of *T*. *cruzi* are heterogeneous process that occurs over time for various cell forms, including the epimastigotes, intermediate forms and metacyclic trypomastigotes in a culture. A representative number of *T*. *cruzi* epimastigote forms are detected during the first hours of *in vitro* metacyclogenesis, even after washing to remove the residual liver infusion tryptose medium (LIT). To address this complexity, the rates at which epimastigotes and metacyclic trypomastigotes are released into the supernatant were followed for 96 h during *in vitro* metacyclogenesis. The analysis of epimastigotes revealed 6.6% epimastigotes in the supernatant 6 to 12 h after the onset of metacyclogenesis, and this rate decreased to 2.5% (p < 0.05) at 96 h. The metacyclic rate was proportionally higher from 24 to 48 h (16.5%, p < 0.001) compared with other time intervals, i.e., from 6 to 12 h (3.1%), 12 to 24 h (6.1%), 48 to 72 h (9.8%, p < 0.05) and 72 to 96 h (5.8%), Fig. 1B.

The number of epimastigotes and metacyclic trypomastigotes in the supernatant allows estimation of the number of parasites that adhered in the flasks, and approximately half the population was found to be adherent until a culture time of 96 h (Fig. 1B). Within this time frame, the time points selected for phospho(proteomic) analysis addressed various adherent time intervals (Ad), with the adherent time points of 24–72 h presenting a significant increase in the metacyclic form for a minor percentage of epimastigote form.

The epimastigotes subjected to nutritional stress for 2 h (St) and purified metacyclic trypomastigotes after 72 h (Mt), were used as experimental control (Fig. S1A). The time point of 6 h was removed from the selected points due to the high degree of epimastigotes remaining in the supernatant, and the time point of 96 h was removed based on the low efficiency of metacyclic release (compared with that observed at 48 and 72 h).

The relative protein and phosphosite quantitation using mass spectrometry was obtained through label free strategy previously processed with FASP protocol and sample fractionation by reverse phase in high pH (Rp HpH) (Fig. S1B and C). After sample separation for proteome run, the remaining samples were enriched for identification of phosphorylated peptides (Fig. S1D). The samples were analysed in biological duplicate for proteome and phosphoproteome, with each technical replicate analysed three times for proteome data and twice for phosphorylation data, to increase the number of protein groups identified (Fig. S1E).

For the data analysis the time points Ad12h and Ad24h were combined and denoted the “*adherent high meta*” (AdhM) due to the high potential of differentiation that was observed. Similarly, the final time points (Ad48h and Ad72h) were also combined, and the combination was denoted “*adherent low meta*” (AdlM) because of the low potential for differentiation into the metacyclic form. These combinations were performed using the expression intensities between points.

### Protein intensity changes during metacyclogenesis

Among the 4,060 protein groups identified in this study (for Proteome data overview go to Supplementary Information), 1,994 were quantified with high confidence using the reporter-ion intensities over the overall analysed time points. Using multiple sample tests, 799 proteins were found to show significant expression regulation throughout metacyclogenesis (Table S5), and 410 of these have putative functions that have been attributed to orthologous comparisons. Fisher’s exact test of GO enrichment terms (Table S6) and clusterization (Fig. S4) were performed to detect the expression profile and orthologous comparison of these proteins.

**Figure 2:**
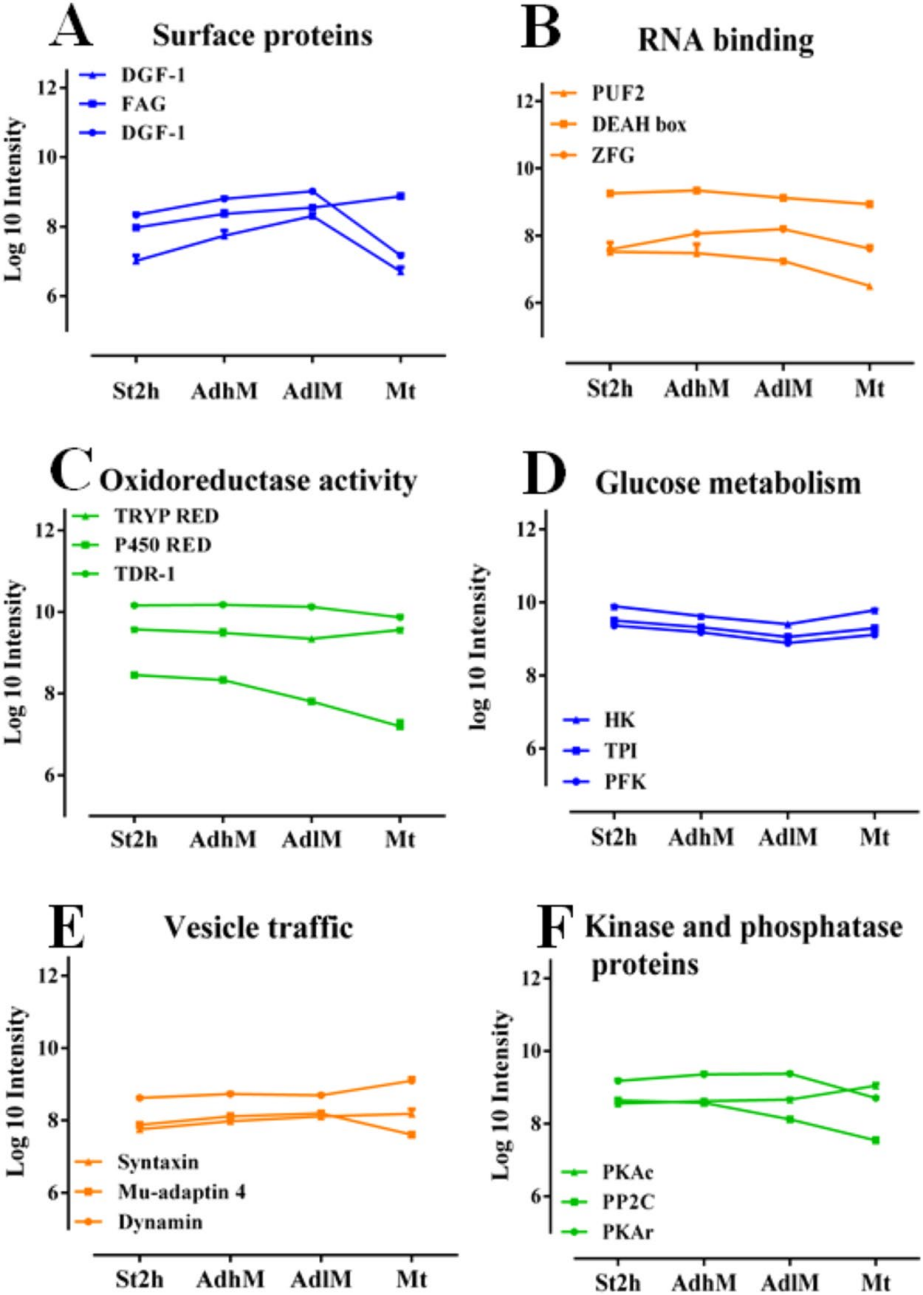
DEPs related to changes in biological processes and molecular functions during metacyclogenesis. (**A**) Surface proteins: disperse gene family 1 protein, DGF-1, *p* value = 2.3E^−7^ and 2.1E^−3^; and flagellar adherent glycoprotein, FAG, *p* value = 2.1E^−4^. (**B**) RNA-binding proteins: Pumilio 2, PUF2, *p* value = 3.8E^−3^; RNA helicase, DEAH box, *p* value = 3.2E^−5^; and zinc finger protein, ZFG, *p* value = 6.2E^−4^. (**C**) Oxidoreductase activity proteins: trypanothione reductase, TRYRED, *p* value = 1.9E^−3^; P450 reductase, P450 RED, *p* value = 2.7E^−7^; and thiol-dependent reductase-1, TDR-1, *p* value = 3.4E^−5^. (**D**) Glucose metabolism-related proteins; hexokinase, HK, *p* value = 9.4E^−4^; 6-phosphofructokinase, PFK, *p* value = 2.0E^−3^; and triosephosphate isomerase, TPI, *p* value = 1.1E^−4^. (**E**) Vesicle traffic-related proteins: dynamin, *p* value = 3.9E^−5^; mu-adaptin 4, *p* value = 1.5E^−4^; and syntaxin, *p* value = 1.5E^−3^. (**F**) Kinase and phosphatase proteins: regulatory protein kinase A-like, PKAr, *p* value = 1.6E^−5^; protein phosphatase 2C, PP2C, *p* value = 3.8E^−5^; and catalytic protein kinase A, PKAc, *p* value = 7.4E^−5^. The expression is presented as the log 10 value of the protein LFQ intensity. The data from two independent biological experiments, each with technical triplicates, were statistically analysed by a multiple-sample test (one-way ANOVA with Benjamini-Hochberg for FDR correction). Values of *p* < 0.01 and q < 0.01 obtained from one-way ANOVA and the FDR control, respectively, were considered to indicate significant differences.

**Figure 3:**
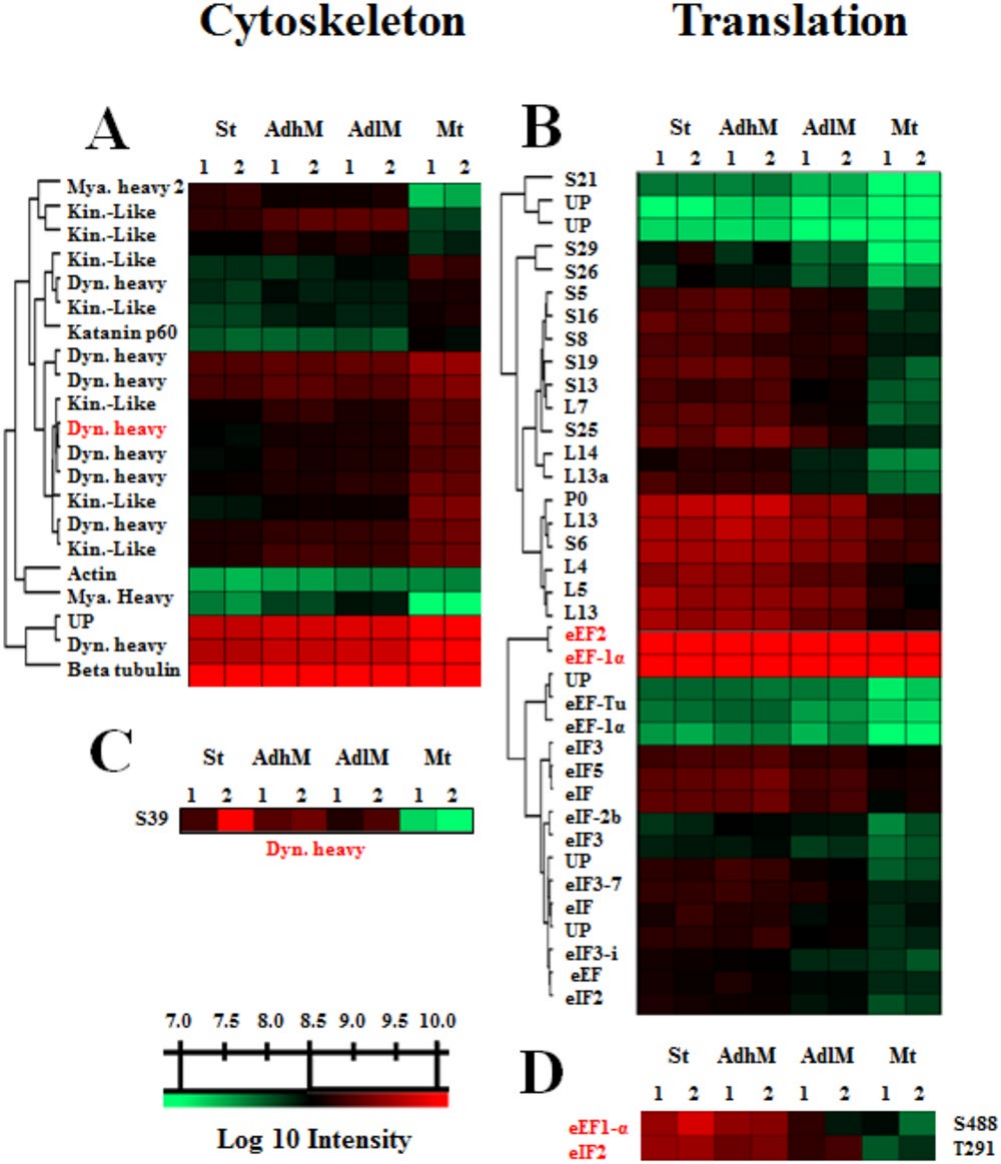
DEPs and DESs related to cytoskeleton component and translation process during metacyclogenesis. (**A**) Cytoskeleton DEPs. (**B**) Translation DEPs. (**C**) Dyn. heavy S39 DES. (**D**) eEF1-α S488 and eEF2 S291 DESs. Heat map of DEPs and DESs during the metacyclogenesis process. The biological time points (columns) and protein groups or phosphosites (lines) were hierarchically clustered, demonstrating expression differences among the stress, adherent, and metacyclic phases. The coloured bar represents the log10 value of the protein or phosphosite intensity. The data from two independent biological experiments (the proteome data were obtained from technical triplicates and the phosphoproteome data were obtained from technical duplicates) were statistically analysed by a multiple-sample test (one-way ANOVA with Benjamini-Hochberg for FDR correction). Values of *p* < 0.01 from one-way ANOVA and values of q < 0.01 (for proteome data) and q < 0.025 (for phosphosite data) obtained from a FDR control were considered to indicate significant differences. 1 and 2 represent the first and second biological replicate for time points. Mya. heavy, myosin heavy chain; kin.like, kinesin-like; katanin p60, Katanin p60 ATPase-containing subunit A1; UP, uncharacterized protein.

The more established changes during metacyclogenesis are related to the cell surface and cytoskeletal alterations^9^. The differentially expressed cell surface proteins identified in this study are represented by trans-sialidases, mucin-associated surface proteins (MASP) and dispersed gene family 1 (DGF-1). The first two groups present more changes during the Mt phase, and the last group is overexpressed during the Ad phases (AdhM and AdlM; Fig. 2A). Flagellum-adhesion glycoprotein (FAG) modulation (Fig. 2A), which was found to progressively increase until Mt, was also observed, reinforcing the importance of adhesion during metacyclogenesis. The analysis of cytoskeletal components showed differential regulations of subpellicular microtubule proteins and motor proteins as well as flagellar components, and these regulations occur at all analysed phases of metacyclogenesis (Fig. 3A).

As demonstrated by Fisher’s exact test, a representative number of differentially expressed events during the process (St and Ad phases) are related to gene expression, and these are mostly included in clusters 1 and 5 (Fig. S4). This biological process includes nuclear events related to the overrepresentation of replication components, such as DNA ligase and topoisomerase. Other activities occur in the cytosol compartment, and these include the control of certain events associated with RNA metabolism. The first is mediated by DEAD-box RNA helicases, some of which are overexpressed during the AdhM and Mt phases. The other three principal classes of RNA-binding proteins (RBPs) are the following: (1) RNA recognition motif (RRM), (2) zinc finger protein, and (3) Pumilio and Fem-3-binding factor (PUF), which is overexpressed primarily in the AdhM phase (Fig. 2B).

The other cytosolic proteins that were modulated during metacyclogenesis are related to translation, oxidoreductase activity and protein metabolism (Fig. S4). The translation-related proteins present a significant number of differentially expressed proteins (ribosomal, initiation and elongation factors) that show higher expression during the St and AdhM phases (Fig. 3B). The sulphur protein groups in oxidoreductase proteins are modulated during the St and Mt phases, whereas the family of haeme proteins include a group with increased expression and another showing a progressive decrease in expression during metacyclogenesis (Fig. 2C).

**Figure 4:**
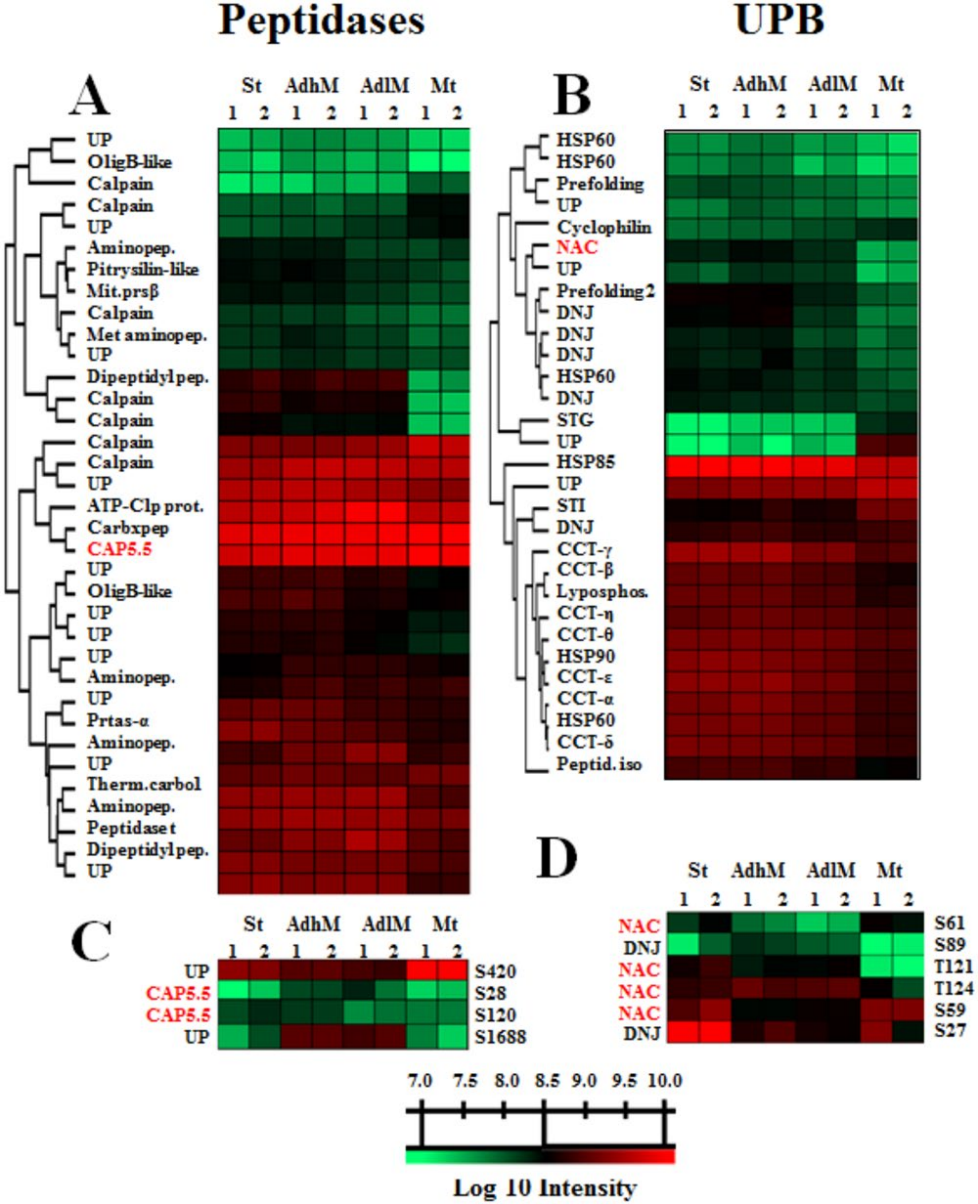
DEPs and DESs related to peptidase activity and unfolded protein binding during metacyclogenesis. (**A**) DEPs with peptidase activity. (**B**) Unfolded protein-binding DEPs. (**C**) CAP5.5 S28 and S120 and UP S420 and S1688 DESs. (**D**) NAC S59, S61, T121 and T124 and DNJ S27 and S89 DESs. Heat map of DEPs and DESs during the metacyclogenesis process. The biological time points (columns) and protein groups or phosphosites (lines) were hierarchically clustered, demonstrating expression differences among the stress, adherent, and metacyclic phases. The coloured bar represents the log10 values of the protein or phosphosite intensity. The data from two independent biological experiments (the proteome data were obtained from technical triplicates, and phosphoproteome data were obtained from technical duplicates) were statistically analysed by a multiple-sample test (one-way ANOVA with Benjamini-Hochberg for FDR correction). Values of *p* < 0.01 from one-way ANOVA and values of q < 0.01 (for proteome data) and q < 0.025 (for phosphosite data) obtained from a FDR control were considered to indicate significant differences. 1 and 2 represent the first and second biological replicate for time points. UPB, unfolded protein binding; UP, uncharacterized protein; OligB-like, oligopeptidase B; Calpain, calpain cysteine peptidase; Aminopep., aminopeptidases; Pitrilysin-like, pitrilysin-like metalloprotease; Mit.prs.β, mitochondrial processing peptidase beta subunit; Met aminopep., methionine aminopeptidase; Dipeptidyl pep., dipeptidyl-peptidase; ATP-Clp pep., ATP-dependent Clp protease subunit; Carbxpep., carboxypeptidase; CAP5.5, cytoskeleton-associated protein CAP5.5; Prtas-α, proteasome alpha 7 subunit; Therm.carbol., thermostable carboxypeptidase 1; STG, small glutamine-rich tetratricopeptide repeat protein; STI, stress-induced protein 1; NAC, nascent polypeptide-associated complex subunit; Peptid.iso., peptidylprolyl isomerase.

The proteins related to protein metabolism, as represented by peptidases and proteins related to unfolded protein binding (UPB), constitute almost 10% of the proteins from four different clusters that are differentially expressed during metacyclogenesis (Fig. S4). The more representative peptidase groups were calpain and aminopeptidases, but the representations of all of the classes (cysteine, metallo-, serine and threonine peptidases) were modulated (Fig. 4A). The main overrepresented UPB are heat shock proteins 60, 70 and 90 (HSP60, 70, 90), small HSPs, peptidylprolyl isomerase, and nascent polypeptide-associated complex subunit (NAC). Co-chaperonins HSP40, small glutamine-rich tetratricopeptide repeat protein (SGT) and stress-induced protein sti1 (STI1) were also detected (Fig. 4B).

The mitochondrial events that were regulated during metacyclogenesis, in addition to some mitochondrial UPB members, are related to amino acid catabolism and its connection to lipid metabolism and oxidative phosphorylation. The largest number of members with the term mitochondria is present in cluster 5 (Fig. S4). Glycosome proteins that are modulated during metacyclogenesis are related to glucose metabolism through glycolytic and pentose phosphate pathways. The first pathway exhibits overexpression during the St and Mt phases, whereas the latter shows a progressive increase until the Mt phase.

These data also show differential expression of vesicle traffic proteins (primarily during the Mt phase; Fig. 2E) and groups of transporters, including the ABC and folate/pteridine transporters (St and AdhM phases). The signalling pathway molecules and cofactors are calmodulin, regulatory subunits of protein kinase A-like (PKAr-like; Fig. 2F, and Fig. S4, cluster 1), adenylate cyclase (Fig. S4, cluster 2), protein phosphatases 2C (PP2C; Fig. 2F, Fig. S4, cluster 3), RAC serine-threonine kinase (Fig. S4, cluster 4); and some proteins in cluster 5. These include the catalytic subunit of protein kinase A (PKAc; Fig. 2F) and protein phosphatase 5 (PPP5), which were also differentially expressed.

### Differential phosphosite modulation during metacyclogenesis

From the 7,336 identified phosphosites (an outline of the phosphoproteome data is included in the Supplementary Information), 1,175 sites were obtained after normalization based on the intensity of the quantified proteins in the proteome dataset and filtering by the high-confidence intensity for all of the samples. Using a multiple-sample test, 260 of these phosphosites were found to be significantly regulated during metacyclogenesis (Fig. S8 and Table S11), and 64 of these have putative functions, as demonstrated from orthologous comparisons with 47 proteins (Table S12).

**Figure 5:**
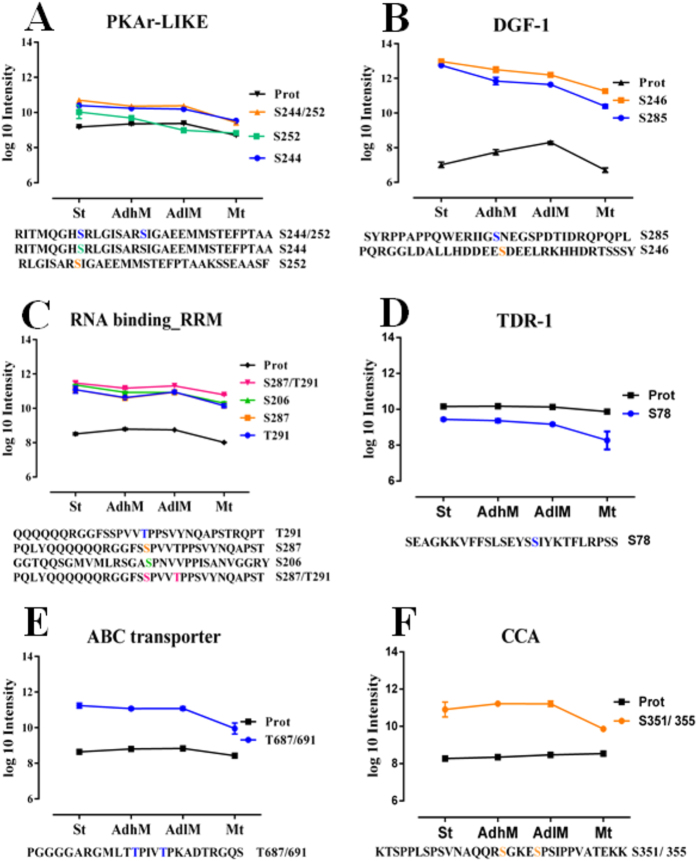
DESs related to changes in biological processes and molecular functions during metacyclogenesis. (**A**) Regulatory protein kinase A-like, PKAr, *p* value = 2.1E^−4^. (**B**) Disperse gene family 1 protein, DGF-1, *p* value = 1.0E^−4^. (**C**) RNA-binding protein with RRM domain, *p* value = 1.1E^−4^. (**D**) Thiol-dependent reductase 1, TDR-1, *p* value = 8.4E^−4^. (**E**) ABC transporter protein, *p* value = 4.0E^−4^. (**F**) Clathrin coat assembly-related protein, CCA, *p* value = 1.1E^−4^. The expression levels are represented as log10 values of the intensity of the protein groups or phosphosites. The bottom parts of each figure represent the phosphosite-containing peptides. The data from two independent biological experiments (the proteome data were obtained from technical triplicates, and phosphoproteome data were obtained from technical duplicates) were statistically analysed by a multiple-sample test (one-way ANOVA with Benjamini-Hochberg for FDR correction). Values of *p* < 0.01 from one-way ANOVA and values of q < 0.01 (for proteome data) and q < 0.025 (for phosphosite data) obtained from a FDR control were considered to indicate significant differences.

The differential phosphorylation throughout metacyclogenesis is broadly distributed across different cell processes, with some of them processing the same differentially expressed proteins in the proteome dataset as the target. One PP2C and two serine/threonine phosphatases A (PP2A) showed differential serine phosphorylation patterns. The PP2C and one of the PP2A exhibited decreased phosphorylation during the process of metacyclogenesis, whereas the other PP2A presented an increase in the phosphorylation during AdhM. The PKAr-like has two phosphosites that are regulated through three phosphorylation states (S244/252, S244, and S252; Fig. 5A).

The nuclear proteins controlled by phosphorylation include transcription activator, nucleoporin 1 (NUP-1) and nucleosome assembly-related protein. The first two of these show significant modulation of serine phosphosites at the St phase, and the latter shows significant modulation during the AdhM phase. The surface protein DGF-1 has two modulated phosphosites called S246 and 285, which show progressive decreases during metacyclogenesis (Fig. 5B). The dynein heavy chain, which has motor function, also demonstrated modulation by phosphorylation, with decreased phosphorylation of S39 in the Mt phase (Fig. 3C).

The cytosolic protein targets of differential phosphorylation that were found to be involved in the translation process were overrepresented in the St and AdhM phases, and these include the following: elongation factor 1-alpha (eEF-1α) and 2 (eEF2) (Fig. 3D). Another cytosolic modulated proteins are RNA metabolism-related proteins [ATP-dependent RNA helicase and RNA-binding proteins (RRM protein with three phosphosites with four phosphorylation states; Fig. 5C)], and oxidative metabolism [thiol-dependent reductase 1 (Fig. 5D) and trypanothione synthetase)].

Of the metabolism-related proteins, cytoskeleton-associated peptidase (CAP5.5) and NAC were modulated. CAP5.5 have multiple phosphorylation sites, and the results showed increased phosphorylation at S28 during the Ad phases and decreased phosphorylation at S120 during metacyclogenesis (Fig. 4C). Similarly, NAC has multiple phosphosites, and S59 and 61 were modulated during the St and Mt phases, whereas T121 and 124 were modulated during the St and Ad phases (Fig. 4D).

Transporters and channels constitute a class of molecules also regulated by phosphorylation on this dataset. Two ABC transporters presented modulation, the first one of which had two sites modulated on T687 and 691 in the St phase (Fig. 5E), while the other one showed modulation on S160 in the AdhM phase. A major vault protein also presented a differentially modulated phosphosite in St, whereas a calcium channel protein showed three modulated sites, two of which (S886 and 890) were modulated simultaneously. Some components involved in vesicle traffic were also found to be controlled by phosphorylation. Beta prime cop protein, transport proteins Sec13 and Rab 6 showed higher phosphorylation rates in the St phase, whereas the Rab 7 and clathrin coat assembly (CCA) proteins presented an increase in phosphorylation during the AdhM phase, this last one at S351/355 (Fig. 5F).

### Analysis of phosphorylation motifs

To correlate the phosphorylation sites with their specific attributed functions and to indirectly infer the affinity of the protein kinases or kinase groups, a search for phosphorylation motifs in all of the identified phosphopeptides throughout metacyclogenesis was conducted. Additionally, to connect the identified motifs and biological processes catalysed by protein kinases, Fischer’s exact test was used for GO terms annotation with the aim of evaluating the overrepresentation of terms.

**Figure 6:**
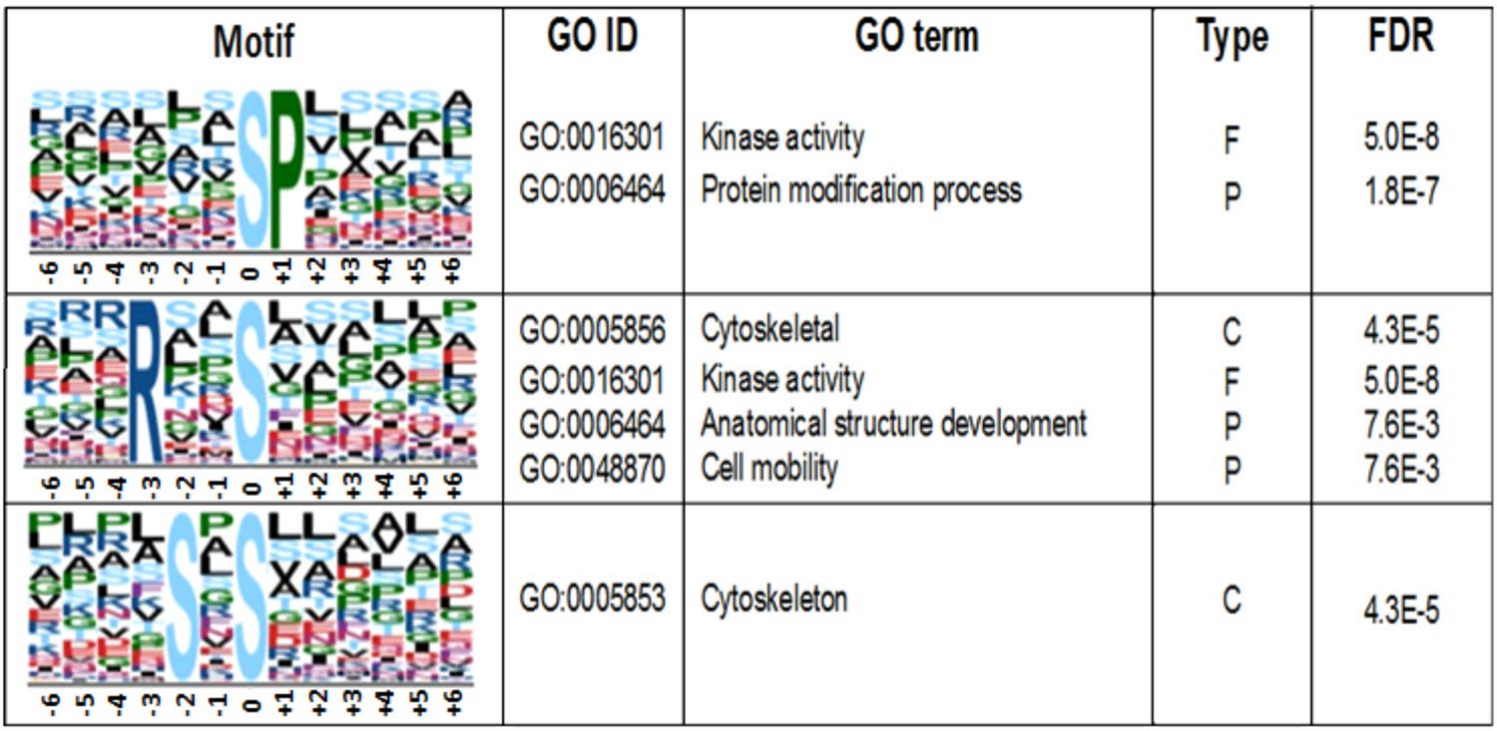
Consensus motif enrichment analysis. Overrepresented phosphorylation motifs among all identified phosphorylation sites compared with all protein coding genes of *T*. *cruzi*. Categorization of GO terms of annotated phosphoproteins containing enriched motifs was determined through Fischer exact test (q < 0.05). The type represents GO enriched terms, P refers to the biological process, F indicates the molecular function, and C represents the cellular component.

Eleven different window-sized datasets ranging from 11 to 31 amino acids around S/T phosphosites extracted 439 motifs from all of the identified sites. Phosphosites containing pY did not demonstrate motif enrichment under the tested conditions. Using 13 amino acids as the window size, 30 and 15 different motifs were found for S and T residues, respectively (for a complete list of motifs, see Table S13) and three of these were associated with enriched GO terms (Fig. 6).

## Discussion

Over the years, different factors have been implicated in influencing the metacyclogenesis of *T*.*cruzi*. Factors such as the oxygen tension, size of the parasite inoculum, age of the culture, parasite strain, tests of several amino acids as the only source of carbon^3^ and the role of adhesion to the substrate^5^ were studied. However, the molecular process regulating the differentiation remains unclear. The heterogeneity of the population during metacyclogenesis generates a considerable difficulty in understanding the process, as has already been observed in other studies^5, 10^. From this biological perspective, our work focused on understanding metacyclogenesis based on the adhered population over time.

The present work presents the greatest coverage of *T*. *cruzi* proteome and the first quantitative phosphoproteome during the metacyclogenesis of the parasite. The importance of the adhesion process was evidenced by the modulation of the FAG, which was already noted in the anterior group’s work^9^. Similarly, the surface and cytoskeleton proteins showed differential expression, as was also observed in the present work. The *T*. *cruzi* surface protein TcDGF-1 was more significantly quantified in the amastigote form compared with the epimastigote and metacyclic forms through antibody development^11^. As demonstrated in this study, the DGF-1 proteins appear to be modulated in the Ad phases, and one of the two proteins is modulated by phosphorylation and presents decreased modification during the process. The increase in the expression of the Ad forms might be related to the presence of adhesion motifs in DGF-1 proteins, this motifs contain segments with significant similarity to human integrin^12^. Cytoskeleton proteins remain among the most representative target members of phosphorylation according to the results of our analysis of phosphorylation motifs, followed by terms such as mobility, kinase activity, and anatomical structure development.

Among the proteins that were differentially expressed during metacyclogenesis are those involved in gene expression, particularly proteins involved in translation, which is considered the primary regulatory mechanism for gene expression control in *T*. *cruzi* ^13^. A few years ago, the importance of phosphorylation-mediated control was elucidated through inhibition of translation leading to *T*. *cruzi* metacyclogenesis. The authors demonstrated that nutritional stress leads to the inhibition of eIF2α, indicating that this stress induces a conserved translation inhibition response in trypanosomatids^7^. Under the conditions tested in this study, eIF2α was not overexpressed on protein or phosphosites level, whereas the eEF1α and eEF2 components of the translation machinery were differentially phosphorylated during the process. This finding might be due to the low expression of eIF2α compared with that of other more abundant proteins, resulting in the inability to detect this molecule.

RBPs have been studied as trans-acting factors during the posttranscriptional control of gene expression in trypanosomatids, particularly due to the accumulation of mRNA granules, as occurs during metacyclogenesis^14^. Immunoprecipitation assays with *T*. *cruzi* RNA helicase (TcDHH1) demonstrate an association with components of stress granules, such as 40S ribosomal subunit proteins, eukaryotic initiation factors 3 and 4 and HSPs^15^, all of which were found to be differentially expressed during metacyclogenesis in the present work. In epimastigotes, Pumilio 6 (TcPUF6) co-localizes with TcDHH1, supporting the hypothesis that TcPUF6 might target mRNAs for degradation, whereas in the metacyclic form, TcPUF6 and TcDHH1 do not colocalize, suggesting that this loss of interaction leads to an increase in associated mRNA^16^. However, the present dataset indicates that in addition to the control mediated by protein expression, some members of RBPs could be regulated by phosphorylation.

The overrepresentation of sulphur and haeme-dependent detoxifying proteins occurs at different stages of the process. This finding is corroborated by the requirement of an antioxidant environment to trigger metacyclogenesis^17^. Trypanothione reductase, an example of a sulphur-modulated protein in the St phase, was found to have its activity inhibited for propyl and isopropyl quinoxaline-derivatives, as was recently established in *T*. *cruzi*, making it a potential drug target^18^. Another sulphur-modulated protein is thiol-dependent reductase 1, which is implicated in deglutathionylation and activation of the antimonial drugs used for treating leishmaniasis^19^. Additionally, thiol-dependent reductase 1 is also modulated by phosphorylation, indicating a possible mechanism for controlling its activities. The analysis of haeme proteins showing modulated profiles throughout metacyclogenesis identified mitochondrial ascorbate-dependent peroxidase, a plant-like ascorbate-dependent peroxidase found in the endoplasmic reticulum of *T*. *cruzi* ^20^. In this context, the significant increase in antioxidant proteins observed in this study is justified by the protein oxidation condition, which is related to increased protein degradation by the proteasome system, as was particularly observed during the 24-h adhesion phase of metacyclogenesis^21^.

The analysis of protein metabolism during metacyclogenesis demonstrated a significant increase in peptidases and UPB, supporting the indication of protein turnover. This control of proteostasis is maintained at the expense of a large number of cysteine, metallo-, serine and threonine peptidases^22^ as well as components of the ubiquitin-proteasome system. The representative members of this entire class of proteins were found to be differentially expressed in the present work, and the last group is highly modulated during the AdhM phase. In *T*. *cruzi*, the proteasome acts as an important mechanism for the control of cell remodelling^23^, and the central machinery is related to the proliferation and differentiation of epimastigotes into the metacyclic trypomastigote form^24^.

UPB include a large number of chaperone and chaperonin proteins. *T*. *cruzi* HSP60, 70 and 90 are involved in various stages of parasite development, particularly in the transition from the metacyclic to amastigote form^25^. The differential expression of cytoplasmatic and mitochondrial HSP40 was observed during *T*. *cruzi* metacyclogenesis. It is known that TcDJ1, a mitochondrial HSP40, shows higher expression levels in epimastigotes compared with the metacyclic form and is possibly involved in mitochondrial biosynthetic processes^26^. TcJ6p, a cytoplasmatic form of HSP40, has significant importance in the early translation event through association with ribosomal proteins^27^. Analysis of the translation process revealed also the NAC overexpression in the AdhM phase, which binds nascent polypeptides in *Saccharomyces cerevisiae* ^28^. However, there is currently no functional description for this protein complex in trypanosomatids. NAC was found to be phosphorylated at four different sites during metacyclogenesis and showed higher levels of phosphorylation during the St phase. Similar to the partnership of HSP40 and HSP70 in *T*. *cruzi* ^29^, SGT appears to interact with HSP70 and HSP90, forming a foldosome inside *Plasmodium falciparum* and facilitating the interaction with client proteins^30^, a process that may also exist in *T*. *cruzi*.

In addition to protein metabolism, the metabolism of amino acids, lipids, and carbohydrates also show modulation during metacyclogenesis. During the AdhM phase, upregulated aminotransferases and transaminases are involved in the release of the amino acid carbon skeleton to supply the citric acid pathway. In addition to serving as a carbon skeleton for other pathways, amino acids are utilized for ATP production, leading to progressive increases in the expression of the alpha and beta subunits of ATP synthase. Lipid metabolism is represented by the differential expression of several enzymes, and of these, fatty acid desaturase, which has been tested as a target for the growth of the *T*. *cruzi* epimastigote form^31^ was found to be overexpressed during the St phase.

Another pathway with members investigated in this study as drug targets for *T*. *cruzi* growth is sterol synthesis^32^, and some of these members were found to be modulated. The analysis of proteins associated with carbohydrate metabolism showed that the differentially expressed proteins are involved in glucose metabolism, among other processes that occur in the glycosome compartment. The glycolytic enzymes were mostly modulated during the St and Mt phases, which indicate that the phosphorylation substrate was used as the preferred energy source during the initial and final phases of metacyclogenesis. Other modulated pathways that occur in the glycosome are the following: (1) gluconeogenesis, which shares enzymes with the glycolytic pathway, (2) the pentose phosphate pathway, and 3) the initial phases of phospholipid metabolism.

The degradation and synthesis of macromolecules to support metabolism and morphological changes are dependent on efficient protein traffic, and in the present dataset, some proteins related to these processes were found to be modulated at the protein and phosphorylation levels. It is known that the endocytosis process occurs late in *T*. *cruzi* metacyclogenesis^33^. Corroborating this requirement, vesicle traffic participants were observed to be overexpressed during the St and AdlM phases. Some of these members are proteins involved in the recognition, fusion, and coating of cargo proteins, as was recently demonstrated for the epimastigote form^34^, but their role in processes such as metacyclogenesis needs to be better studied.

The transporters that show differential expression during metacyclogenesis include a folate/pteridine transporter, which is modulated during the Ad phases, indicating a great need to obtain these molecules (folate and pteridine) during the adhesion phase. *T*. *cruzi* is auxotrophic for both molecules and therefore must acquire them from the host. The addition of biopterin (a pteridine derivative) to *T*. *cruzi* cultivation enhanced the proliferation of epimastigotes under chemically defined conditions^35^, indicating the importance of the input of pteridine for this parasite. Another family of transporters that was found to be overexpressed at the protein level and modulated by phosphorylation in these data is the ABC transporters, which are capable of performing haeme transmembrane transport in *T*. *cruzi* ^36^. The role of the haeme molecule as a cofactor of TcK2 kinase was recently demonstrated to serve as a trivial process in the control of *T*. *cruzi* metacyclogenesis through TceIF2-alpha phosphorylation^8^. Thus, in the absence of haeme, the activation of TcK2 was observed, leading to an increase in metacyclogenesis^8^.In fact, the phosphorylation of ABC transporters might play a major role in controlling the availability of haeme and thereby mediating kinase activity.

One of the most relevant events in differentiation is the identification of molecules responsible for the process of intracellular signalling in response to environmental changes. In this context, kinases, phosphatases, and related molecules also demonstrated differential expression during all analysed phases of metacyclogenesis. The progressive increase of adenylate cyclase until the Mt phase, as well as the modulation of PKAr-like and PKAc during the process, corroborate the results of other studies that reported the role of these molecules in regulating the life cycle of *T*. *cruzi* ^9, 37^. The regulatory subunit of PKAr-like contains two phosphosites that are regulated during metacyclogenesis through three states of phosphorylation. This finding represents an elegant example of the dynamic regulation of different sites in the same protein and the affording of different functions to the same protein^38^. Examples of phosphatases modulated at the phosphorylation level include *T*. *cruzi* phosphatase 2 (TcPP2A), which is involved in the differentiation of trypomastigotes to amastigotes under axenic conditions^39^.

Here, MS-based proteomics and phosphorylated peptide enrichment were conducted to investigate *T*. *cruzi* metacyclogenesis highlighting the adherent population. The *in vitro* metacyclogenesis profile allowed classification of the adhered forms in AdhM and AdlM according to higher and lower differentiation capacities, respectively. The results revealed that molecules such as FAG and DGF-1 are likely involved in the adhesion process because their modulation occurs in the Ad phases. The phospho(proteome) indicated the occurrence of major modulations at the St and AdhM phases. The primary cellular processes that were found to be modulated were translation, oxidative stress, and the metabolism of protein, lipids, and carbohydrates as well as cell signalling triggered by kinases and phosphatases. Many of these processes have already been elucidated to be modulated during metacyclogenesis. However, the present work increases the number of members involved in each process. Also for the first time the phospho(proteome) profile of the different adhered population forms of *T*. *cruzi* was conducted. To the best of our knowledge, this study provides the first quantitative results on the modulation of phosphorylation sites during metacyclogenesis. Many of the members that were found to be modulated in the present work have already been studied as targets in trypanosomiase treatment; however, many alternative possibilities have become proposed. In addition to targets in treatments, proteins and modulated phosphorylation sites are available in databases for future characterizations, such as the identification of partners in protein complexes, point mutations on the modulated phosphosites and gene knockouts.

## Methods

### *In vitro* metacyclogenesis

For the *in vitro* metacyclogenesis of the *T*. *cruzi Dm*28c clone^4^, epimastigotes in the exponential phase of growth cultivated on LIT were used from an initial inoculum of 1 × 10^6^ without shaking. After reaching a density of 5 × 10^7^ parasites/mL (late exponential phase), parasite samples were collected by centrifugation at 3000 × *g* and 20 °C for 10 min. To study nutritional stress, 5 × 10^8^ epimastigotes/ mL were washed once with TAU medium (190 mM NaCl, 17 mM KCl, 2 mM MgCl_2_, 2 mM CaCl_2_, and 8 mM phosphate buffer, pH 6.0), incubated at 28 °C in the same medium and collected after 2 h (St2h). The cultures were then diluted to 5 × 10^6^ parasites/mL in TAU3AAG medium (TAU medium supplemented with 10 mM L-proline, 50 mM L-sodium glutamate, 2 mM L-sodium aspartate, and 10 mM D-glucose) and allowed to adhere (300-cm^2^ culture flasks, TPP, St. Louis, MO, USA). Subsequently, the parasites were collected after 12 h (Ad12h), 24 h (Ad24h), 48 h (Ad48h) and 72 h (Ad72h) by removing the supernatant, washing twice and vigorously shaking the flasks to release the adhered parasites. Metacyclic trypomastigotes (Mt72h) were obtained using a previously described method^40^.

To monitor the differentiation rate, differential counts were performed over 6-96 h of metacyclogenesis in a Neubauer chamber. The data were analysed statistically by two-way analysis of variance (two-way ANOVA) and Tukey’s test for the comparison of averages.

### NanoLC-MS/MS acquisition

Mass spectrometry data were acquired at the mass spectrometry facility RPT02H of Carlos Chagas Institute, Fiocruz, Parana (Sections titled “Protein extraction and digestion” and “Peptide fractionation and phosphopeptide enrichment” are included in the Supplementary Information). Each sample was separated by online reversed-phase nanoscale capillary liquid chromatography and analysed by ESI-MS/MS with an Easy-nLC 1000 UHPLC (Thermo Scientific, Waltham, MA, USA) coupled to an LTQ Orbitrap XL system (Thermo Scientific, Waltham, MA, USA). Chromatographic separation of the peptide mixtures was performed on a fused silica emitter (in a 30 cm length column of 75 µm inner diameter) that was in house packed with reversed-phase ReproSil-Pur C18-AQ 1.9-µm resin (Dr. Maisch GmbH, Ammerbuch-Entringen, Germany).

For the proteome data, the sample were separated at a flow rate of 250 nL/ min using 0.5% formic acid and 5% DMSO over a linear gradient from 5% to 28% MeCN for 207 min and from 28% to 40% MeCN over 23 min (total gradient of 240 min). The phosphopeptides enriched samples were separated over a linear gradient from 5% to 40% MeCN over 120 min. DMSO was used to improve the electrospray ionization of the peptides^41^. Mass spectra were acquired in the positive-ion mode by applying a data-dependent automatic survey MS scan in the tandem mass spectra (MS/MS) acquisition mode. The generated peptides were eluted from each LC run, detected as three-dimensional features (retention time versus signal intensity-extracted ion chromatogram (XIC) versus mass/charge) and consecutively compared across runs.

Full-scan MS spectra (at 300–1650 *m/z* range) were acquired on an Orbitrap analyser with a resolution of R = 60,000 (after the accumulation of a target value of 1,000,000 using the preview mode). The seven (phosphoproteome) or 10 (proteome) most intense ions were sequentially isolated and fragmented by collision-induced dissociation (CID) in the linear ion trap at a target value of 30,000. The MSA CID was selected for the phosphoproteome. The general conditions for the MS were as follows: spray voltage of 2.3 kV; no cover and auxiliary gas flow; ion transfer tube temperature of 175 °C, and normalized collision energy of 35% for MS2. The ion selection thresholds were 100 counts for MS2. An activation q of 0.25 and an activation time of 30 ms were applied to the MS2 acquisitions. The “lock mass” option was enabled (m/z = 401.922718) for all the full scans to improve the mass accuracy of the precursor ion^42^.

### Data availability

The mass spectrometry phospho(proteome) data have been deposited to the ProteomeXchange Consortium under accession number PXD006171. The complete procedure used for the data analysis is included in the Supplementary Information.

## Electronic supplementary material

Supplementary information

Table S1.

Table S2.

Table S3.

Table S4.

Table S5.

Table S6.

Table S7.

Table S8.

Table S9.

Table S10.

Table S11.

Table S12.

Table S13.

## References

[1] Chagas, C. Nova tripanozomiase humana. Estudos sobre a morfolojía e o ciclo evolutivo de Schizotrypanum cruzin. gen., n. sp., ajente etiolójico de nova entidade morbida do homen. *Mem*. *Inst*.*Oswaldo Cruz* 159–218 (1909).

[2] World Health Organization. Chagas Disease. *Chagas Disease* Available at: http://www.who.int/mediacentre/factsheets/fs340/en/ (Accessed: 2nd April 2017) (2017).

[3] Contreras VT, Salles JM, Thomas N, Morel CM, Goldenberg S (1985). *In vitro* differentiation of Trypanosoma cruzi under chemically defined conditions. Mol. Biochem. Parasitol..

[4] Contreras V (1988). Biological aspects of the DM28c clone of Trypanosoma cruzi after metacyclogenesis in chemically denined media. Mem. Inst.Oswaldo Cruz.

[5] Bonaldo MC, Souto-Padron T, de Souza W, Goldenberg S (1988). Cell-substrate adhesion during Trypanosoma cruzi differentiation. J. Cell Biol..

[6] Figueiredo RC, Rosa DS, Soares MJ (2000). Differentiation of Trypanosoma cruzi epimastigotes: metacyclogenesis and adhesion to substrate are triggered by nutritional stress. J. Parasitol..

[7] Tonelli RR, Augusto S, Castilho BA, Schenkman S (2011). Protein Synthesis Attenuation by Phosphorylation of eIF2 alpha Is Required for the Differentiation of Trypanosoma cruzi into Infective Forms. PLoS One.

[8] da Silva Augusto L (2015). A Membrane-bound eIF2 Alpha Kinase Located in Endosomes Is Regulated by Heme and Controls Differentiation and ROS Levels in Trypanosoma cruzi. PLoS Pathog..

[9] de Godoy LMF (2012). Quantitative proteomics of Trypanosoma cruzi during metacyclogenesis. Proteomics.

[10] Ferreira LRP, Dossin FDEM, Ramos TC, Schenkman S (2008). Active transcription and ultrastructural changes during Trypanosoma cruzi metacyclogenesis. An. Acad. Bras. Cienc..

[11] Lander N (2010). Localization and developmental regulation of a dispersed gene family 1 protein in Trypanosoma cruzi. Infect. Immun..

[12] Kawashita SY, da Silva CV, Mortara RA, Burleigh BA, Briones MRS (2009). Homology, paralogy and function of DGF-1, a highly dispersed Trypanosoma cruzi specific gene family and its implications for information entropy of its encoded proteins. Mol. Biochem. Parasitol..

[13] Smircich P (2015). Ribosome profiling reveals translation control as a key mechanism generating differential gene expression in Trypanosoma cruzi. BMC Genomics.

[14] Cassola A, De Gaudenzi JG, Frasch AC (2007). Recruitment of mRNAs to cytoplasmic ribonucleoprotein granules in trypanosomes. Mol. Microbiol..

[15] Holetz FB (2010). Protein and mRNA content of TcDHH1-containing mRNPs in Trypanosoma cruzi. FEBS J..

[16] Dallagiovanna B (2008). Functional genomic characterization of mRNAs associated with TcPUF6, a pumilio-like protein from Trypanosoma cruzi. J. Biol. Chem..

[17] Nogueira NP (2015). Proliferation and differentiation of Trypanosoma cruzi inside its vector have a new trigger: Redox status. PLoS One.

[18] Chacón-Vargas K (2017). Trypanocidal Activity of Quinoxaline 1,4 Di-N-oxide Derivatives as Trypanothione Reductase Inhibitors. Molecules.

[19] Fyfe PK, Westrop GD, Silva AM, Coombs GH, Hunter WN (2012). Leishmania TDR1 structure, a unique trimeric glutathione transferase capable of deglutathionylation and antimonial prodrug activation. Proc. Natl. Acad. Sci. USA.

[20] Wilkinson SR, Obado SO, Mauricio IL, Kelly JM (2002). Trypanosoma cruzi expresses a plant-like ascorbate-dependent hemoperoxidase localized to the endoplasmic reticulum. Proc. Natl. Acad. Sci. USA.

[21] Cardoso J (2011). Analysis of proteasomal proteolysis during the *in vitro* metacyclogenesis of Trypanosoma cruzi. PLoS One.

[22] Alvarez VE, Niemirowicz GT, Cazzulo JJ (2012). The peptidases of Trypanosoma cruzi: Digestive enzymes, virulence factors, and mediators of autophagy and programmed cell death. Biochim. Biophys. Acta - Proteins Proteomics.

[23] Diego JLD (2001). The Ubiquitin - Proteasome Pathway Plays an Essential Role in Proteolysis during Trypanosoma cruzi Remodeling. Biochemistry.

[24] Cardoso J (2008). Inhibition of proteasome activity blocks Trypanosoma cruzi growth and metacyclogenesis. Parasitol. Res..

[25] Paba J (2004). Proteomic analysis of the human pathogen Trypanosoma cruzi. Proteomics.

[26] Carreira MAC (1998). TcDJ1, a putative mitochondrial DnaJ protein in Trypanosoma cruzi. FEMS Microbiol. Lett..

[27] Salmon D, Montero-Lomelí M, Goldenberg S (2001). A DnaJ-like Protein Homologous to the Yeast Co-chaperone Sis1 (TcJ6p) Is Involved in Initiation of Translation in Trypanosoma cruzi. J. Biol. Chem..

[28] Ott AK, Locher L, Koch M, Deuerling E (2015). Functional dissection of the nascent polypeptide-associated complex in Saccharomyces cerevisiae. PLoS One.

[29] Edkins AL, Ludewig MH, Blatch GL (2004). A Trypanosoma cruzi heat shock protein 40 is able to stimulate the adenosine triphosphate hydrolysis activity of heat shock protein 70 and can substitute for a yeast heat shock protein 40. Int. J. Biochem. Cell Biol..

[30] Hatherley R, Clitheroe CL, Faya N, Tastan Bishop Ö (2015). Plasmodium falciparum Hop: Detailed analysis on complex formation with Hsp70 and Hsp90. Biochem. Biophys. Res. Commun..

[31] Alloatti A, Testero SA, Uttaro AD (2009). Chemical evaluation of fatty acid desaturases as drug targets in Trypanosoma cruzi. Int. J. Parasitol..

[32] Kessler RL, Soares MJ, Probst CM, Krieger MA (2013). Trypanosoma cruzi Response to Sterol Biosynthesis Inhibitors: Morphophysiological Alterations Leading to Cell Death. PLoS One.

[33] Vidal JC, Alcantara C, de L, de Souza W, Cunha-e-Silva NL (2016). Loss of the cytostome-cytopharynx and endocytic ability are late events in Trypanosoma cruzi metacyclogenesis. J. Struct. Biol..

[34] Kalb LC (2016). Conservation and divergence within the clathrin interactome of Trypanosoma cruzi. Sci. Rep..

[35] De Paula Lima CV (2014). LM14 defined medium enables continuous growth of Trypanosoma cruzi. BMC Microbiol..

[36] Cupello MP (2011). The heme uptake process in Trypanosoma cruzi epimastigotes is inhibited by heme analogues and by inhibitors of ABC transporters. Acta Trop..

[37] Gonzales-Perdomo M, Romero P, Goldenberg S (1988). Cyclic AMP and adenylate cyclase activators stimulate Trypanosoma cruzi differentiation. Exp. Parasitol..

[38] Olsen JV (2006). Global, *in vivo*, and site-specific phosphorylation dynamics in signaling networks. Cell.

[39] González J (2003). A novel protein phosphatase 2A (PP2A) is involved in the transformation of human protozoan parasite Trypanosoma cruzi. Biochem. J..

[40] Sousa MAde (1983). A simple method to purify biologically and antigenically preserved bloodstream trypomastigotes of Trypanosoma cruzi using DEAE-cellulose columns. Mem. Inst. Oswaldo Cruz.

[41] Hahne H (2013). DMSO enhances electrospray response, boosting sensitivity of proteomic experiments. Nat. Methods.

[42] Schroeder M, Shabanowitz J (2004). A neutral loss activation method for improved phosphopeptide sequence analysis by quadrupole ion trap mass spectrometry. Anal. Chem..

