# Supplementary material for: Quantitative proteome and phosphoproteome analyses highlight the adherent population during Trypanosoma cruzi metacyclogenesis
Source: Sci Rep. 2017 Aug 29;7:9899. doi: 10.1038/s41598-017-10292-3 (PMC5574995; doi:10.1038/s41598-017-10292-3)
Supplement: Supplementary file 1 — Supplementary information [file 41598_2017_10292_MOESM1_ESM.pdf]

Supplementary information

Quantitative proteome and phosphoproteome analyses highlight the adherent population during *Trypanosoma cruzi* metacyclogenesis

Juliana C. Amorim[1], Michel Batista[1, 2], Elizabeth S. da Cunha[1], Aline C. R. Lucena[1], Carla V. de Paula Lima[1], Karla Sousa[1], Marco A. Krieger[1], Fabricio K. Marchini[1,2*]

[1] Functional Genomics Laboratory, Carlos Chagas Institute, Fiocruz, Curitiba, Parana, Brazil

[2] Mass Spectrometry Facility - RPT02H, Carlos Chagas Institute, Fiocruz, Curitiba, Parana, Brazil

*__Corresponding Author:__ fabricio.marchini@fiocruz.br.

Supplementary Results

Proteome data overview

Global identification of the proteome dataset was extracted from 149 LC-MS/MS runs (two biological replicates, with four fractions from each and three LC-MS/MS runs for each fraction; five replicates were added due to the low anterior coverage), and 2,007,811 MS/MS spectra (corresponding to 39.4% of the total acquired) addressed 31,852 non-redundant peptide sequences belonging to 4,060 protein groups (**Tables S1, S2, and S3**).

The data obtained during metacyclogenesis are robust with respect to reproducibility, as demonstrated by a Pearson correlation coefficient among replicates of each time point that is higher than 0.98 (**Figure S2A**). The principal component analysis (PCA) revealed that the protein expression patter presented intermediate forms at Ad12h, Ad24h, Ad48h, and Ad72h more closely related to each other and to St than to Mt (**Figure S2B**).

One of the typical events in the Kinetoplastea class is a large number of proteins with uncharacterized functions. These organisms diverged early in the eukaryote domain, reflecting low sequence similarity among proteins. From the total protein groups identified here, 2,379 (58.6%) were annotated as uncharacterized proteins, reflecting the high proportion of genes with unknown functions in the *T. cruzi* genome and their significance during the differentiation process in the present scenario (**Table S3**).

In relation to previous work that addressed the general metacyclogenesis process through a proteome approach, the present study focused on the adherent populations and their progressive changes. The comparative analysis of the data confirmed that more than 1,300 proteins groups were not previously detected and the present data cover 99.6% of previously described proteins. The remaining protein groups that are not present in our dataset can be

explained by unanalysed biological forms (epimastigotes in the exponential phase of growth and those that were subjected to stress for 30 min; **Figure S2C**).

An analysis using the GO and KEGG terms associated with all of the proteins identified during metacyclogenesis through Fisher's exact test revealed an enriched process compared with all Tc*Dm*28c protein-coding genes, making the differentiation event more evident (**Figures S3A and S3B and Table S4**). Some examples were the overrepresentation of cell differentiation, cell morphogenesis and cytoskeleton organization terms, which are expected to occur during metacyclogenesis. However, the cell adhesion, carbohydrate and lipid metabolism processes are underrepresented, as demonstrated based on the identified proteins, and this result was unexpected because cell adhesion has been established as an essential event in metacyclogenesis. In addition, important metabolic pathways are dependent on carbohydrate polymers and lipid molecules, particularly under differentiation conditions. From these results, the analysis described in the main text (**Protein intensity changes during metacyclogenesis**) provided an understanding of which processes, functions, and cellular components are differentially expressed during differentiation, contributing to the elucidation of essential quantitative changes throughout metacyclogenesis.

Outline of phosphoproteome data

The combined data from the phosphopeptide-enriched samples were obtained from a total of 96 LC-MS/MS runs (two biological replicates, with four fractions from each and two LC-MS/MS runs for each fraction), and 357,527 MS/MS spectra were identified (equivalent to 36.4% of the total acquired), corresponding to 9,374 non-redundant peptide sequences belonging to 2,859 protein groups, with a false discovery rate of 1% (**Tables S8, S9, and S10**).

In total, 7,336 phosphorylation sites on 2,041 phosphoprotein groups were identified with considerable correlation and becoming quantitative compared to anterior work,

published by us in 2011 (**Figure S5A, Table S10**). The previous work identified 2,572 phosphosites, with an overlap of 1,470 phosphosites with the present dataset. With this, the current work presents more than 5,900 exclusive sites (**Figure S5B**). This difference might be due to the methodology employed as well as the parasite populations at different stages of differentiation. A PCA of phosphosites intensity, as was observed for proteome data, revealed that the patters of expression of the adhered populations (Ad) are more similar to St than to Mt (**Figure S5C**).

The localization probability for the phosphogroup within a phosphopeptide with single-amino-acid accuracy was determined using the PTM score obtained with MaxQuant. The results demonstrate that the majority of the positions could be determined with high confidence ($\geq$ 0.99 = 3,888 sites; 53%; **Figure S6A**).

The majority of the 4,805 unique phosphopeptide determinations, specifically, 2,489 (51.8%), were singly phosphorylated, whereas up to 306 peptides (6.4%) showed multiple phosphorylated sites (more than four modified sites per peptide; **Figure S6B**). These results are consistent with those obtained in a previous study, which showed a TiO_2 microbeads bias to preferentially bind monophosphorylated peptides. The phosphorylated amino acid residue distributions during the *T. cruzi* metacyclogenesis process were 5,538 pS (75.46%), 1,692 pT (23.06%) and 106 pY (1.44%) (**Figure S6C**).

The number of phosphorylated tyrosine residues detected in this work increased more than four-fold compared with previously reported findings (106 versus 26 pY), whereas complete identification increased approximately three-fold. Tyrosine phosphorylation plays an important role in cell signalling and the differentiation process, and in the present dataset, some classes of protein groups with tyrosine phosphorylation were identified. Among these proteins, mitogen-activated protein kinase, calcium channel proteins, calcium-binding

proteins and several serine/threonine protein kinases were found to be phosphorylated on tyrosine residues.

Compared with all Tc*Dm*28c protein-coding genes, the phosphoproteins identified during metacyclogenesis demonstrate prevalent terms through GO and KEGG annotations. Of the cellular components, the cilium and nuclear pore are representative terms. Calmodulin and phospholipid binding, as well as kinase activity, are molecular functions with a significant number of members. The representative terms for biological processes are the glucose metabolic process and protein transport (**Figure S7A, Figure S8, Table S9**). In addition, the metabolic pathways with the highest number of representatives identified were the pentose phosphate pathway and the biosynthesis of unsaturated fatty acids (**Figure S7B)**.

Similar to the proteome data approach, the following analyses were conducted to understand which phospho-regulated processes are differentially expressed during metacyclogenesis, as described in the main text (**Differential phosphosite modulation during metacyclogenesis**).

Supplementary Methods

Protein extraction and digestion

Protein extraction: At each time point during differentiation (St2h, Ads12-72h and Mt72h), 10 mg of total protein (corresponding to $6x10^9$ for the parasite metacyclic form and $3x10^9$ for the other parasite forms) was extracted with lysis buffer (0.1 M Tris-HCl, pH 7.6, 0.1 M DTT and 4% SDS), incubated at 95 °C for 5 min and sonicated with 30% duty cycle and output control 3 (Cole-Parmer 500-Watt Ultrasonic Homogenizer, Vernon Hills, IL, USA). The remaining debris was removed by centrifugation at 16,000 × *g* and 4 °C for 10 min, and the supernatants were collected and stored at -80 °C until use.

Protein purification and digestion: For protein purification, the FASP protocol was used with some modifications. In brief, an Amicon Ultra-15 centrifugal filter unit 10 K (Merck-Millipore, Darmstadt, Germany) containing 5 mg of protein concentrate was incubated with washing solution (6.5 mL of 8 M urea, 0.1 M Tris-HCl, pH 8.5 and 0.01 M DTT). The samples were then centrifuged at 4,000 x g and 20 °C for 45 min, and this step was repeated three times. Subsequently, 3.25 mL of 0.05 M iodoacetamide was diluted in the washing solution and incubated in the dark for 20 min, and the samples were then centrifuged under the above-mentioned conditions. After protein alkylation, two subsequent washes were performed with washing solution. Afterward, 3.25 mL of 0.05 M ammonium bicarbonate (ABC) solution was added to the filters, and the samples were centrifuged; this step was repeated once. Trypsin was added at 1:100 (w/w enzyme to substrate ratio) and diluted in 1.5 mL of 0.05 M ABC solution for each filter, and the samples were incubated for 18 h at 37 °C. The peptides were then collected by two sequential centrifugations at 4,000 x g for 40 min, first with 1 mL of 0.05 M ABC and then with 1 mL of 0.5 M NaCl. The peptide samples were then quantified with a NanoDrop spectrophotometer (Thermo Scientific, Waltham, MA, USA) at an absorbance of 280 nm using an extinction molar coefficient of 1100 and a peptide mass average of 1400 Da.

Peptide fractionation and phosphopeptide enrichment

Peptide fractionation: To remove salt and reduce sample complexity, high pH reversed-phase fractionation using a 130-mg Sep-Pak C18 Plus Light Cartridge (Waters, Milford, MA, USA) was performed. In brief, 95 mL of ammonium formate (AF) at pH 10 (0.08% v/v ammonium hydroxide from Sigma with 0.016% v/v formic acid from Merck) was added to the total peptide elution (approximately 5 mL). The peptides (0.5 mg) were added to a pre-equilibrated Sep-Pak column and washed twice with 1 mL of 20 mM AF. The elution procedure was performed serially using 1 mL of 20 mM AF combined with 10, 14, 18 or 60% acetonitrile (MeCN) from Merck. The peptide fractions were acidified with 0.5%

trifluoroacetic acid (TFA) from Merck and quantified in the NanoDrop as previously described. Approximately 10% of the peptides from each fraction was desalted in C18 StageTips and resuspended in 0.1% formic acid, 5% DMSO, and 5% MeCN prior to analysis by LC-MS/MS.

Phosphopeptide enrichment: For a short time, 0.5 mg of peptides from each fraction were dried in a vacuum centrifuge and suspended in 1 mL of loading solution (2.5% TFA, 80% MeCN, 70 mg/ mL phthalic acid from Sigma). The peptide solution was then added to 2 mg of pre-equilibrated 10 μm TiO_2 microbeads (GL Science, Saitama, Japan) with end-over-end rotation for 60 min. The microbeads were washed twice with 500 μL of loading solution, twice with 500 μL of wash buffer A (80% MeCN, 0.1% TFA), and twice with 500 μL of wash buffer B (0.1% TFA). The peptides were eluted sequentially three times with 25 μL of 0.3 M ammonium hydroxide solution. They were then dried in a vacuum centrifuge, purified with C18 StageTips and resuspended in 0.1% formic acid, 5% DMSO, and 5% MeCN prior to LC-MS/MS analysis.

Data analysis

Data processing was performed using the Andromeda algorithm included in the MaxQuant platform (version 1.5.2.8). The proteins and phosphorylation sites were identified by an automatic search against 11,346 protein sequences derived from the Tc*Dm*28c genome (downloaded in April 2016 from UniProtKB). Contaminant proteins (*Achromobacter lyticus* lysyl endopeptidase, human keratins, BSA and porcine trypsin) and the reverses of all the sequences, including contaminants, were also added to the database, and when identified, these were manually removed from the list of results.

The search parameters specified an MS spectra accuracy of 20 ppm (first search) and 4.5 ppm (second search), a CID MS/MS spectra accuracy of 0.5 Da, and full trypsin specificity, allowing up to two missed cleavages. The carbamidomethylation of cysteine was

set as a fixed modification, and the oxidation of methionines and the N-terminal acetylation of proteins were set as variable modifications. For the phosphoproteomic search, the phosphorylations of serine, threonine, and tyrosine (STY) were also allowed as variable modifications. Peptides were required to be at least seven amino acids in length, proteins had at least two peptides, and a 1% FDR (which was calculated by the number of reverse hits from the searches against the decoy database) was applied at the peptide, protein and phosphorylation site levels. A localization probability ≥ 0.75 and a delta score > 5 were used as criteria for selecting the phosphosites for quantification. Optional matching between runs was enabled to transfer identifications across replicate experiments. The fractions were grouped in the same experimental design to produce a complete list of proteins for each sample. After identification, similar protein sequences in the database that could not be distinguished by the experimentally detected peptides were grouped and referred to as protein groups.

The MaxQuant output was analysed using Perseus version 1.5.5.3, through hierarchical clusterization, Pearson correlation, Venn diagram analysis, PCA, Gene Ontology and KEGG pathway terms of protein and phosphosite intensities. Blast2GO 4.0 version was used for Fisher's exact test. Sequence motif enrichment analyses of the phosphorylation sites were performed using the Motif-X algorithm, with windows from 11 to 31 amino acids. The same *T. cruzi* database that was employed previously for the protein and phosphosite identification was used as the background. For another analysis was used GraphPad Prism version 6 software and the Figures were edited using CorelDRAW graphic suite version X6. The intensity values [the protein group LFQ intensity and phosphosite intensity (containing one, two or three phospho groups)] for each fraction was subjected to log10 transformation.

Only proteins and phosphosites with intensity values for all of the samples were selected for the statistical analysis of differential expression. The differentially expressed

proteins (DEPs) and phosphosites (DESs) were identified by comparing the LFQ and STY intensity values by a multiple-sample test (one-way ANOVA corrected by the Benjamini-Hochberg method to control the FDR). Normalization between phosphopeptide-enriched and non-enriched intensities was achieved through the equation log10 intensity (PhosphoSTY/ProtLFQ) + 12.

Supplementary Figures and Legends

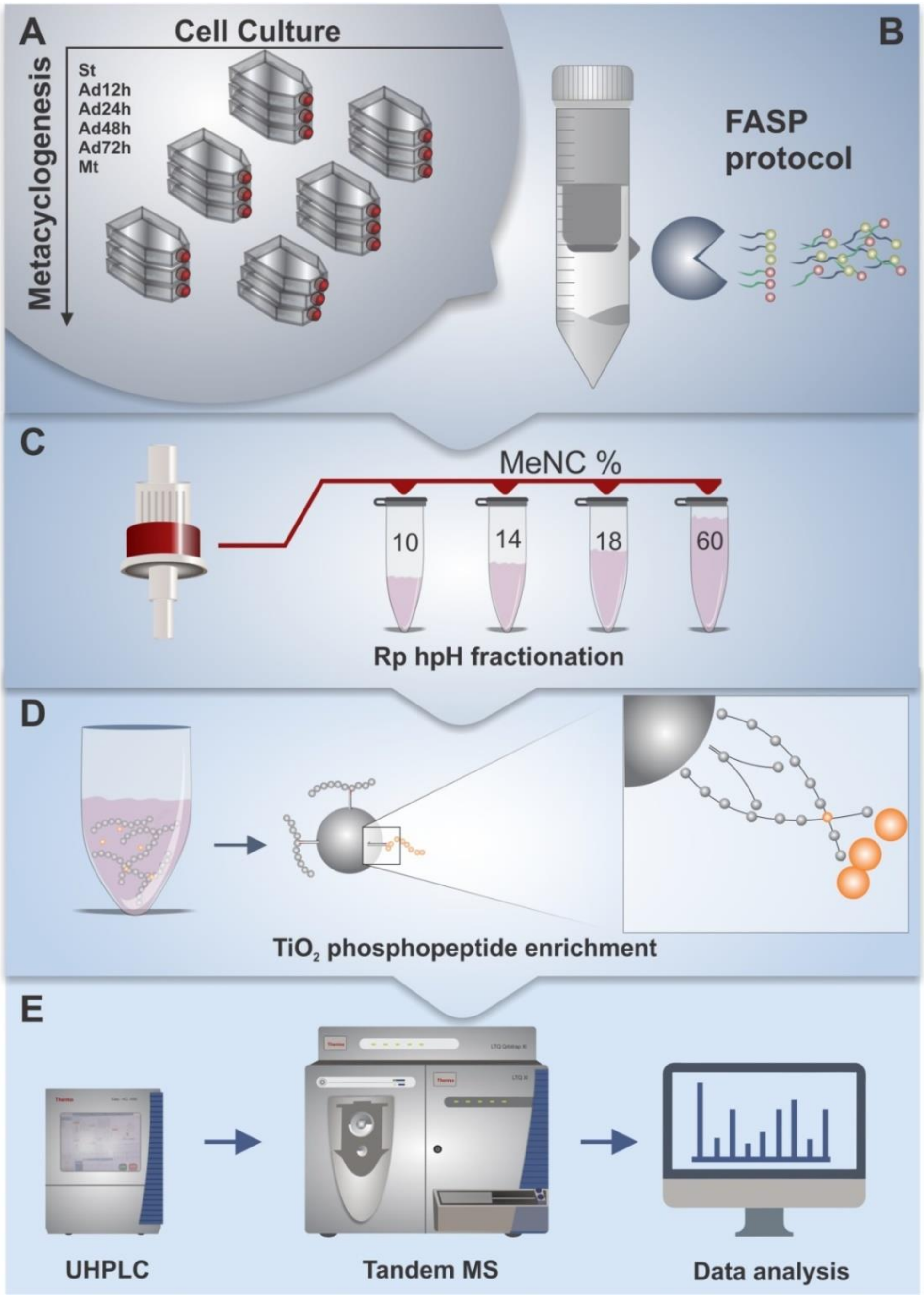

Figure S1. Experimental workflow. (A) *In vitro* metacyclogenesis of *T. cruzi* using TAU for the stress process (St2h) and TAU3AAG for metacyclogenesis over 72 h (Ad12-72h and Mt72h). (B) Sample preparation: the cells collected from the above differentiation time points were lysed in SDS buffer, and the protein extract was purified and digested using the FASP protocol. (C) The samples were separated by reserve phase high pH (Rp hpH) into four fractions using Sep-Pak columns. (D) For complete phosphoproteome identification, the phosphopeptides were enriched by TiO_2 microbeads. (E) LC-MS/MS analysis: all the fractions were separated on a reversed-phase ultra-high performance liquid chromatography (UHPLC) and submitted to electrospray ionization into an LTQ-Orbitrap XL mass spectrometer, which was operated in the data-dependent mode. The protocol details are shown in the methods section.

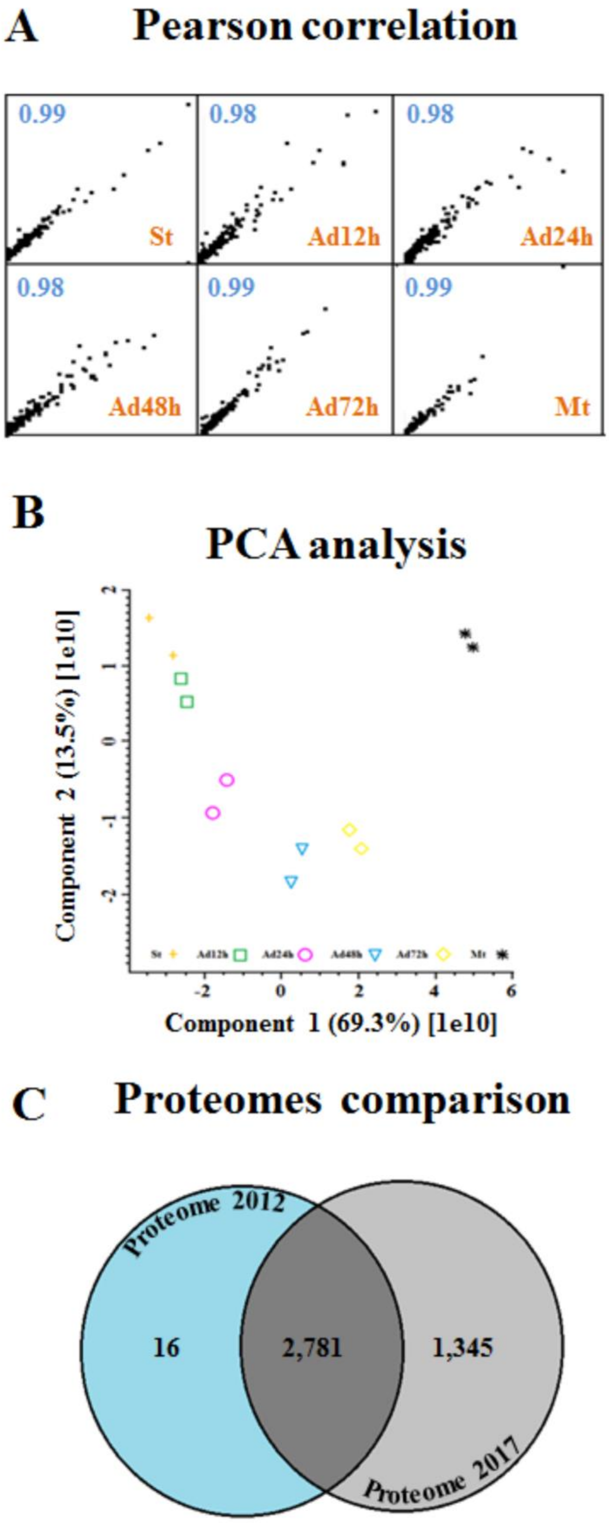

Figure S2. **Global analysis of proteome dataset** (A) The correlation of the log 10 value of the protein LFQ intensity from the proteome dataset between each biologically independent duplicate was determined through a Pearson correlation analysis. (B) The PCA plot of the log 10 value of the protein LFQ intensity from the proteome dataset among biologically independent replicates was determined through multiple comparisons using the FDR (Benjamini-Hochberg, $q < 0.05$). (C) Venn diagram of the comparison of proteomes obtained at 2012 and 2017.

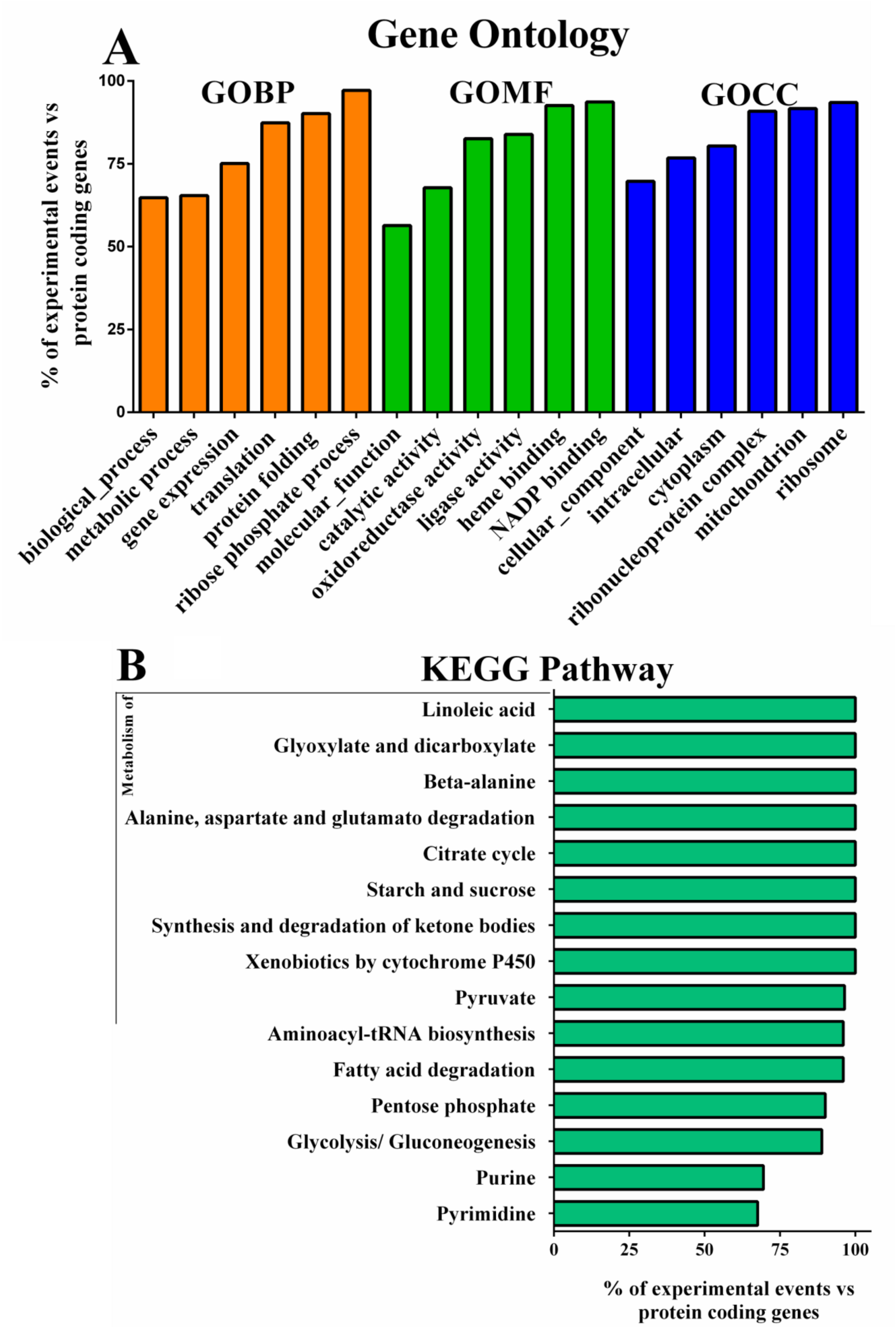

Figure S3. **Gene Ontology and KEGG pathway terms of the identified proteins during *T. cruzi* metacyclogenesis.** (A) GOBP, GOMF, and GOCC are terms for biological process, molecular function and cellular localization according to the GO Slim name, respectively. (B) KEGG pathway term using KEGG Slim name. The analysis was determined through Fischer exact test ($p < 0.05$ and $q < 0.01$). The non-exclusive classification corresponds to the proteins localized in more than one cellular compartment or that participate in more than one biological process or molecular function.

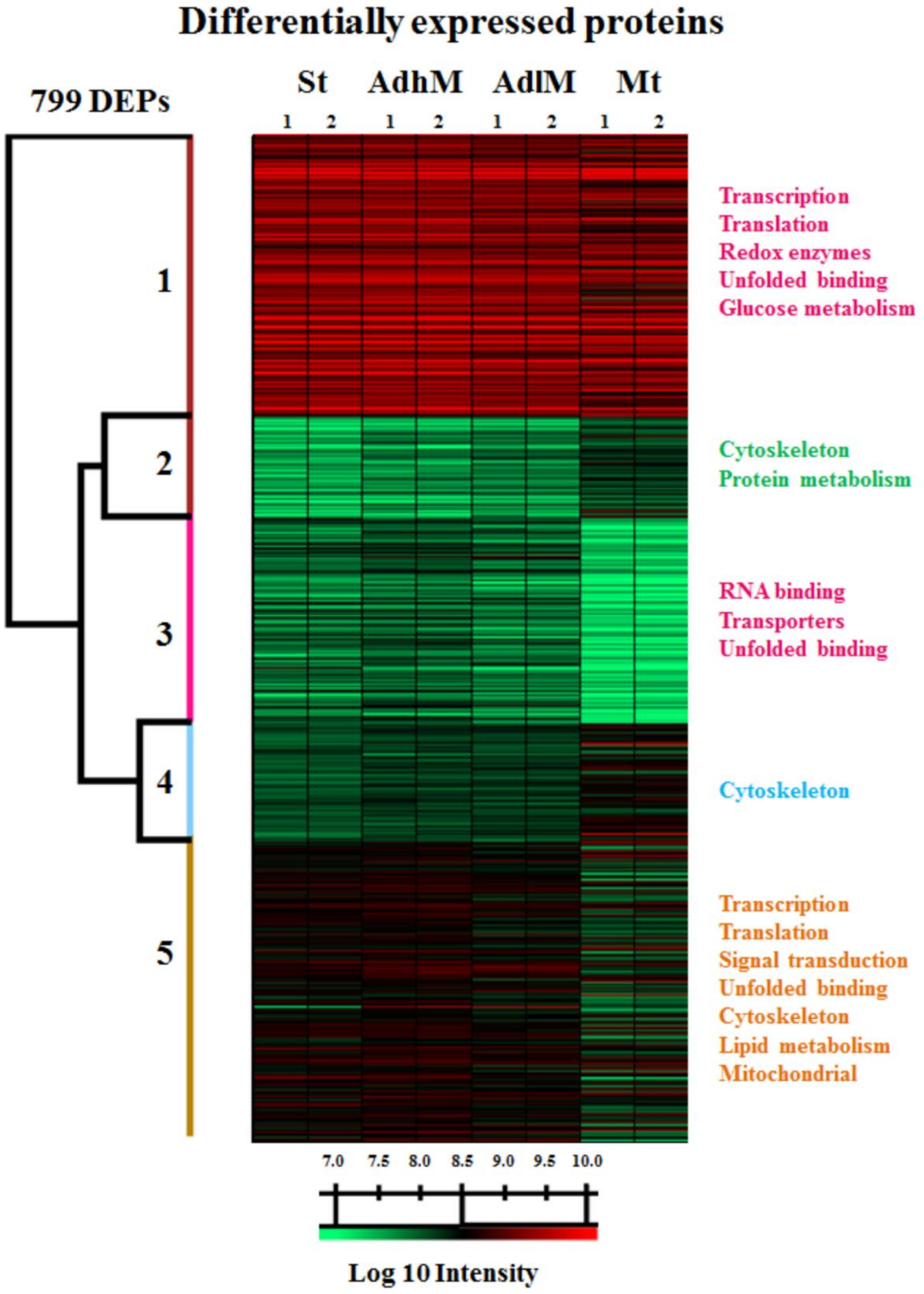

Figure S4. Differentially expressed proteins during metacyclogenesis. All differentially expressed proteins were determined statistically by multiple-sample test (one-way ANOVA supported by FDR using Benjamini-Hochberg) of two independent biological experiments, each of one was performed in technical triplicate. The results indicate 799 proteins groups (lines) through time points (columns) represented as heat maps of five clusters, the $p < 0.01$, and q <0.01 was considered significantly differentiated, respectively for one-way ANOVA and FDR control. 1 and 2 represent the first and second biological replicate for time points. The coloured bar represents the log 10 values of the protein LFQ intensity. The parameters for clustering were Euclidean distance, with five clusters and ten maximal interactions.

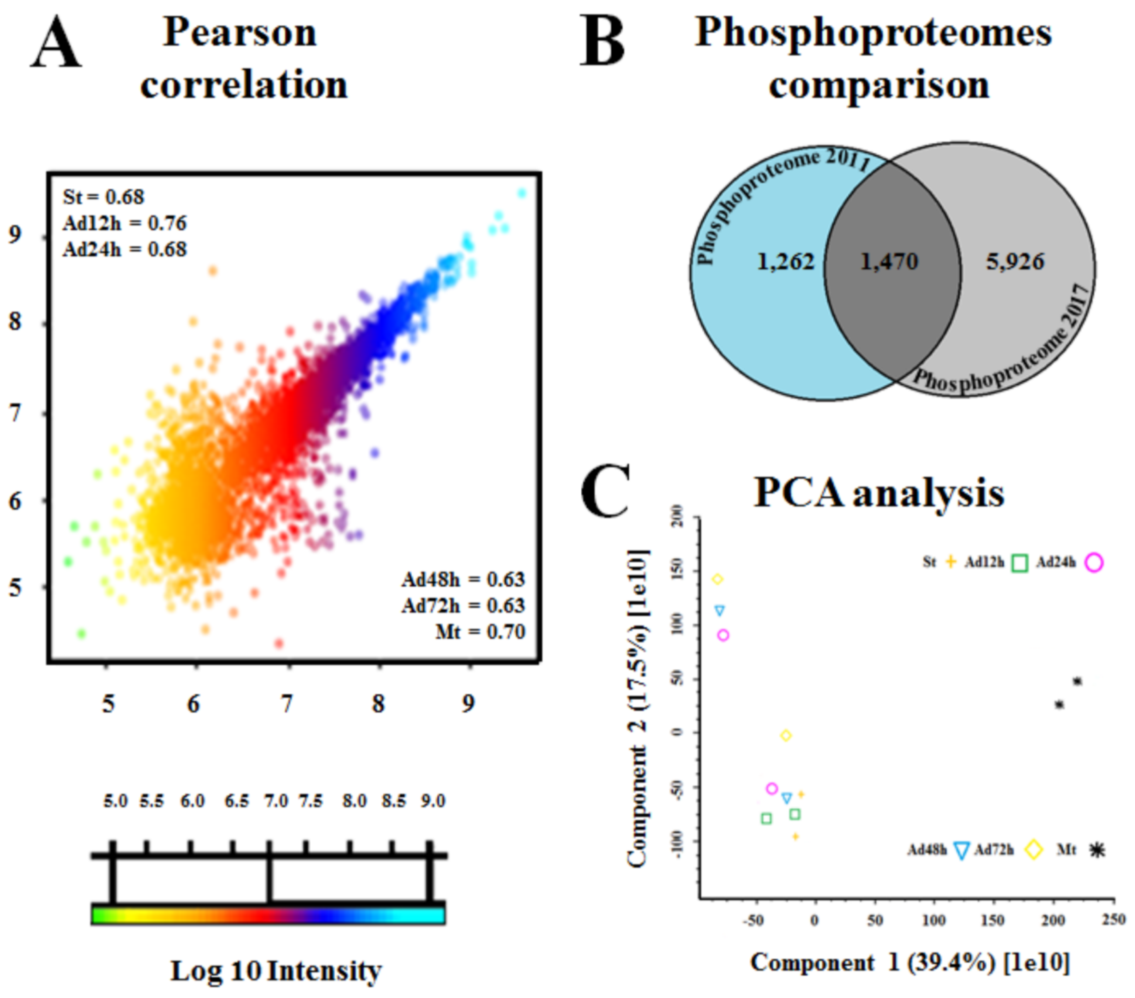

Figure S5. General phosphoproteome data obtained during metacyclogenesis. (A) The correlation of the log 10 value of the phosphosite intensity from the non-normalized phosphoproteome dataset between each biologically independent duplicate was determined through a Pearson correlation analysis (over) and using the colour-based intensity (under). (B) The PCA plot of the log 10 value of the phosphosite intensity from the non-normalized phosphoproteome dataset among biologically independent replicates was determined through multiple comparisons using the FDR (Benjamini-Hochberg, q < 0.05). (C) Venn diagram of the comparison of the phosphoproteomes obtained in 2011 and 2017.

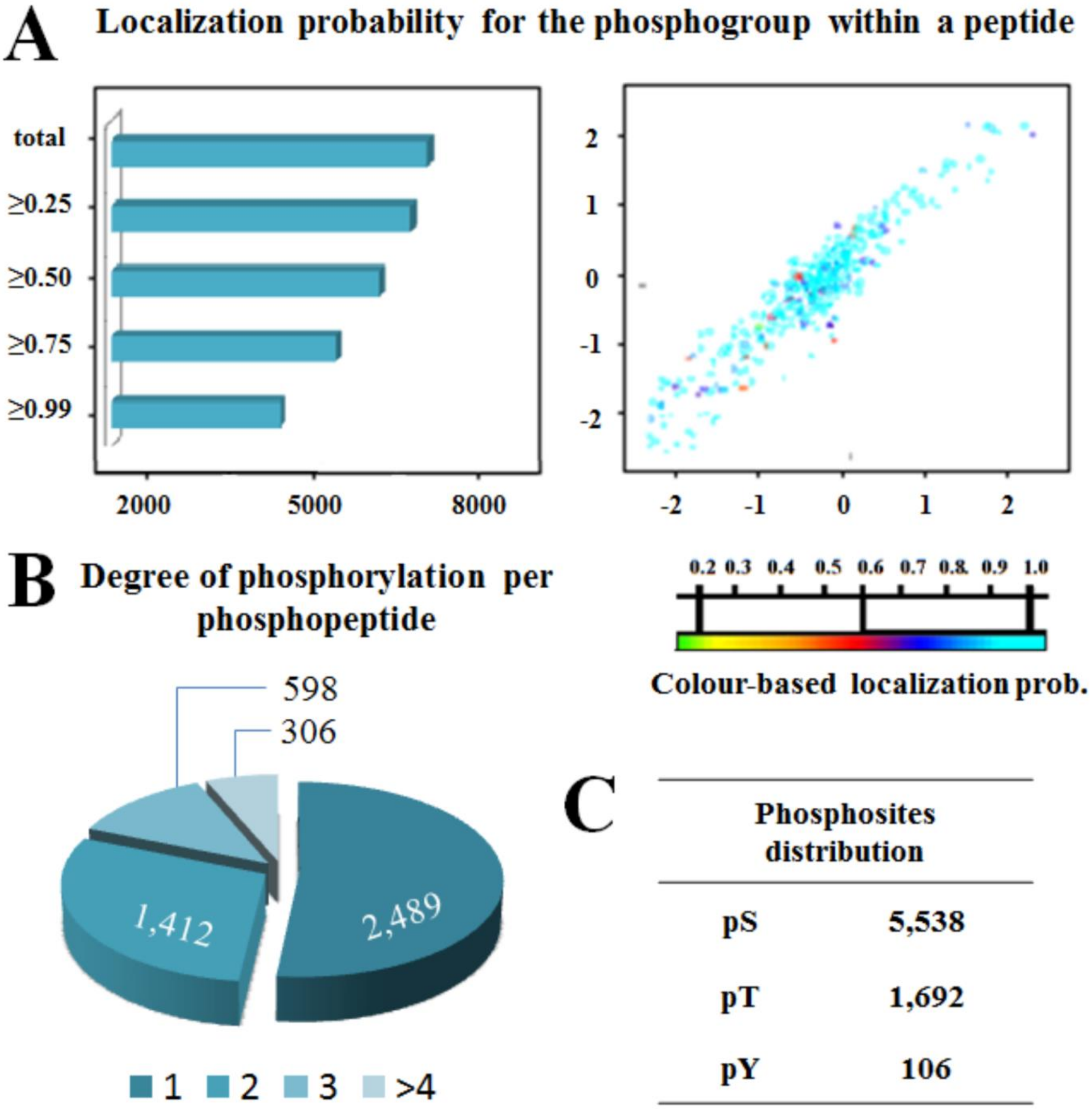

Figure S6. General phosphopeptide and phosphosite data obtained during metacyclogenesis. (A) The localization probability of the phosphogroups within a phosphopeptide based on the localization probability ≥ 0.99, ≥ 0.75, ≥ 0.5, and ≥ 0.25 probability (left) and, colour-based localization probability (right). (B) The degree of phosphorylation per phosphopeptide: 1, 2, 3 and 4 represent the numbers of phosphosites per phosphopeptide. (C) Phosphosite distribution on Ser (pS), Thr (pT), and Tyr (pY).

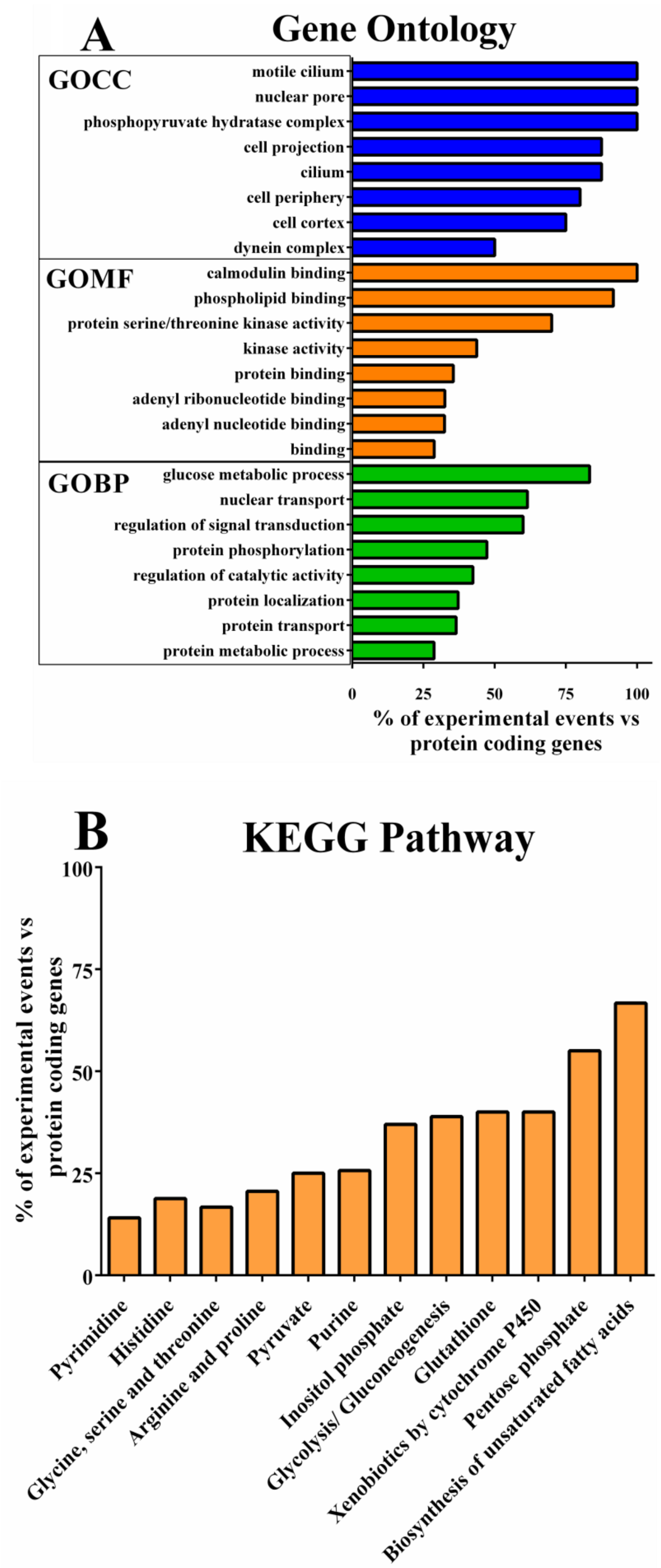

Figure S7. Gene Ontology and KEGG pathway terms of the identified phosphoproteins during *T. cruzi* metacyclogenesis (A) GOBP, GOMF, and GOCC are terms for biological process, molecular function and cellular localization, according to the GO Slim name, respectively. (B) KEGG pathway term using the KEGG Slim name. The analysis was determined through Fischer exact test ($p < 0.05$ and $q < 0.025$).The non-exclusive classification corresponds to the proteins localized in more than one cellular compartment or that participate in more than one biological process or molecular function.

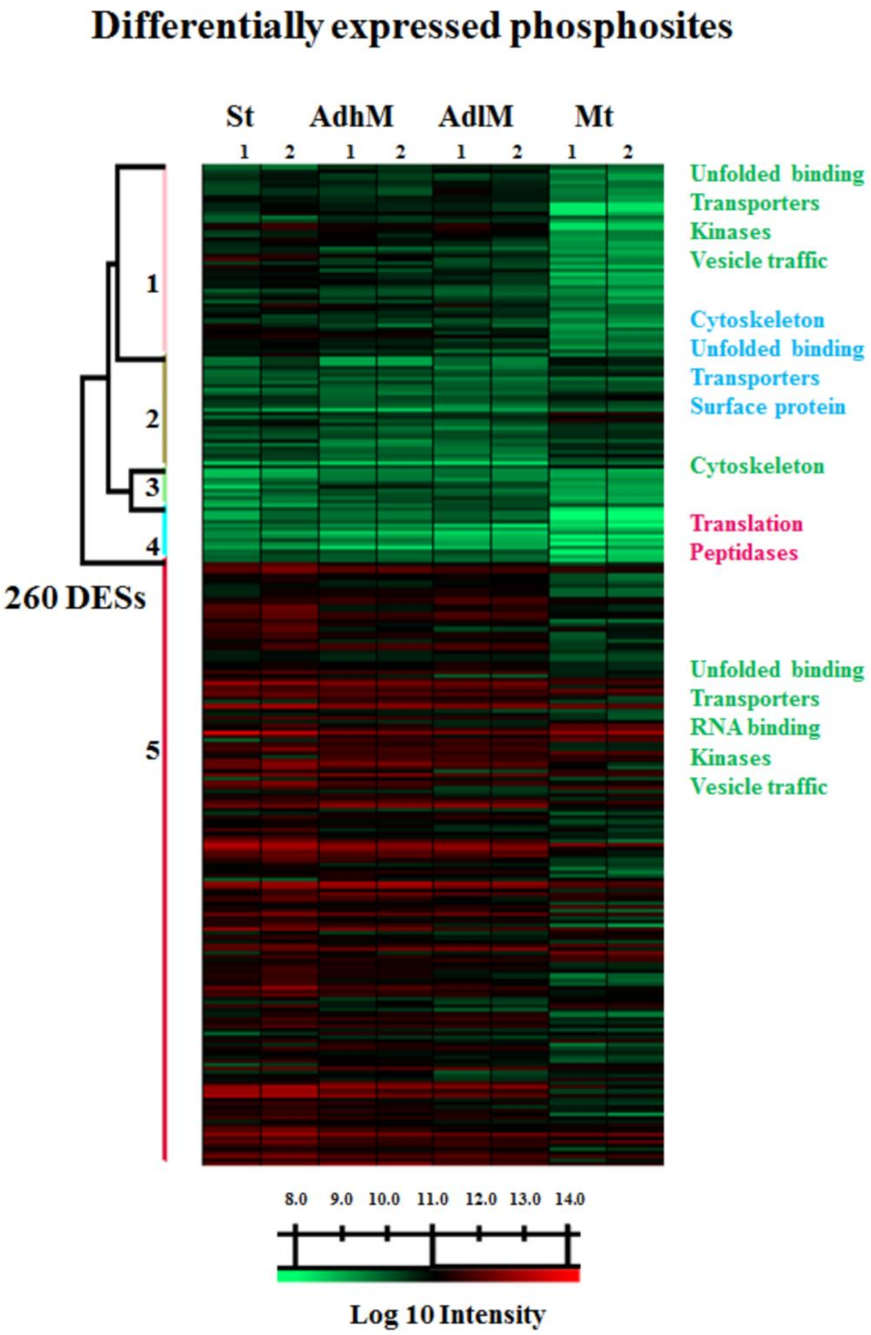

Figure S8. Differentially expressed phosphosites during metacyclogenesis. All differentially expressed phosphosites were determined statistically by multiple-sample test (one-way ANOVA supported by FDR using Benjamini-Hochberg) of two independent biological experiments, each of one was performed in technical duplicate. The results indicate 260 phosphosites (lines) through time points (columns) represented as heat maps of five clusters, the $p < 0.01$, and q <0.025 was considered significantly differentiated, respectively for one-way ANOVA and FDR test. 1 and 2 represent the first and second biological replicate for time points. The coloured bar represents the log 10 values of the phosphosite intensity. The parameters for clustering were Euclidean distance, with five clusters and ten maximal interactions.

Supplementary Tables

Table S1. Summary parameters used for *T. cruzi* metacyclogenesis proteome dataset acquisition.

Table S2. Description of each peptide identified during *T. cruzi* metacyclogenesis proteome dataset acquisition.

Table S3. Description of each protein group identified and quantified during *T. cruzi* metacyclogenesis proteome dataset acquisition.

Table S4. GO enriched terms after applying Fischer's exact test ($p < 0.01$, $q < 0.01$) for all identified proteins during *T. cruzi* metacyclogenesis versus all protein-coding genes.

Table S5. Description of each DEP during *T. cruzi* metacyclogenesis proteome dataset acquisition after applying a multiple-sample test (one-way ANOVA and Benjamini-Hochberg for controlling the FDR) with $p < 0.01$ and $q < 0.01$.

Table S6. GO enriched terms after applying Fischer's exact test ($p < 0.01$, $q < 0.01$) for all DEPs during *T. cruzi* metacyclogenesis versus all protein-coding genes.

Table S7. Summary parameters used for *T. cruzi* metacyclogenesis phosphoproteome dataset acquisition.

Table S8. Description of each phospho(peptide) identified during *T. cruzi* metacyclogenesis phosphoproteome dataset acquisition.

Table S9. Description of each phospho(protein group) identified and quantified during *T. cruzi* metacyclogenesis phosphoproteome dataset acquisition.

Table S10. Description of each phosphorylation site identified during *T. cruzi* metacyclogenesis phosphoproteome dataset acquisition.

Table S11. Description of each DES during *T. cruzi* metacyclogenesis phosphoproteome dataset acquisition after applying a multiple-sample test (one-way ANOVA and Benjamini-Hochberg for controlling the FDR) with $p < 0.01$ and $q < 0.025$.

Table S12. Putative DESs during *T. cruzi* metacyclogenesis categorized as a biological process and molecular function.

Table S13. Phosphorylation motifs obtained with a window size of 13 amino acids using Sp or Tp as the central character and $p < 10^6$ as the significance cutoff.

Competing Interests: The authors declare that they have no competing interests.
